# Global miRNA/proteomic analyses identify miRNAs at 14q32 and 3p21, which contribute to features of chronic iron-exposed fallopian tube epithelial cells

**DOI:** 10.1038/s41598-021-85342-y

**Published:** 2021-03-18

**Authors:** Ravneet Chhabra, Stephanie Rockfield, Jennifer Guergues, Owen W. Nadeau, Robert Hill, Stanley M. Stevens, Meera Nanjundan

**Affiliations:** 1 Department of Cell Biology, Microbiology, and Molecular Biology, University of South Florida, Tampa, FL 33620 USA; 2 Department of Pharmaceutical Sciences, Albany College of Pharmacy and Health Sciences, 261 Mountain View Drive, Colchester, VT 05446 USA; 3 Department of Cell and Molecular Biology, St. Jude Children’s Research Hospital, 262 Danny Thomas Place, Memphis, TN 38105 USA

**Keywords:** Biological techniques, Cancer, Cell biology, Pathogenesis

## Abstract

Malignant transformation of fallopian tube secretory epithelial cells (FTSECs) is a key contributing event to the development of high-grade serous ovarian carcinoma (HGSOC). Our recent findings implicate oncogenic transformative events in chronic iron-exposed FTSECs, including increased expression of oncogenic mediators, increased telomerase transcripts, and increased growth/migratory potential. Herein, we extend these studies by implementing an integrated transcriptomic and mass spectrometry-based proteomics approach to identify global miRNA and protein alterations, for which we also investigate a subset of these targets to iron-induced functional alterations. Proteomic analysis identified > 4500 proteins, of which 243 targets were differentially expressed. Sixty-five differentially expressed miRNAs were identified, of which 35 were associated with the “top” proteomic molecules (> fourfold change) identified by Ingenuity Pathway Analysis. Twenty of these 35 miRNAs are at the 14q32 locus (encoding a cluster of 54 miRNAs) with potential to be regulated by DNA methylation and histone deacetylation. At 14q32, miR-432-5p and miR-127-3p were ~ 100-fold downregulated whereas miR-138-5p was 16-fold downregulated at 3p21 in chronic iron-exposed FTSECs. Combinatorial treatment with methyltransferase and deacetylation inhibitors reversed expression of these miRNAs, suggesting chronic iron exposure alters miRNA expression via epigenetic alterations. In addition, PAX8, an important target in HGSOC and a potential miRNA target (from IPA) was epigenetically deregulated in iron-exposed FTSECs. However, both PAX8 and ALDH1A2 (another IPA-predicted target) were experimentally identified to be independently regulated by these miRNAs although TERT RNA was partially regulated by miR-138-5p. Interestingly, overexpression of miR-432-5p diminished cell numbers induced by long-term iron exposure in FTSECs. Collectively, our global profiling approaches uncovered patterns of miRNA and proteomic alterations that may be regulated by genome-wide epigenetic alterations and contribute to functional alterations induced by chronic iron exposure in FTSECs. This study may provide a platform to identify future biomarkers for early ovarian cancer detection and new targets for therapy.

## Introduction

Iron is essential for maintenance of cellular homeostasis and organismal survival^1^. Iron participates in Fenton reactions, yielding reactive oxygen species (ROS) that are highly damaging to macromolecules including proteins, lipids, and nucleic acids^2^. Increased intracellular iron accumulation is a key feature of ferroptosis, a programmed cell death mechanism that is characterized by increased lipid peroxidation^3,4^. In contrast, deregulated expression of mediators involved in iron metabolism (i.e., Transferrin Receptor) leads to increased intracellular labile iron that promotes increased cellular proliferative capacity and cancer pathogenesis^5,6^. In our prior report, in response to supraphysiological levels of NTBI (non-transferrin bound iron, presented as ferric ammonium citrate (FAC)), we performed a proteomics screen utilizing reverse phase protein array (RPPA) to identify global proteomic alterations, which confirmed RAS- and MAPK-dependency in short-term iron-exposed ovarian cancer cell lines^7,8^.

High-grade serous ovarian cancer is the deadliest gynecological malignancy in women for which the fallopian tube secretory epithelial cell (FTSEC) type is now considered an established precursor cell^9^. Our recent findings have identified that chronic iron exposure contributes to upregulated EVI1 (a transcriptional regulator, amplified at 3q26.2) and TERT expression, accompanied by increased cell numbers and migration in FTSECs^10^. However, to our knowledge, an assessment of global alterations induced by this mode of iron exposure in FTSECs has yet to be performed and is thus necessary to identify comprehensive cellular changes for which improved treatment regimens may be designed. Furthermore, since miRNAs elicit both oncogenic and tumor suppressive functions by altering the expression of multiple protein targets, a global miRNA screening approach in chronic iron exposed FTSECs is deemed to be an additional highly valuable experimental approach.

Herein, we utilized a multiomics approach using the GeneChip miRNA 4.1 microarray and mass spectrometry-based proteomics to identify deregulated miRNAs and protein targets, respectively, in chronic iron exposed and transformed FTSECs. We applied stringent statistical approaches to identify the most significantly deregulated hits and integrated both of these analyses using Ingenuity Pathway Analysis (IPA). A majority of the identified downregulated miRNAs are located at the 14q32 locus, a highly aberrant chromosomal region in multiple tumor types^11–13^. Since 14q32 miRNAs can be regulated by differentially methylated promoter regions (i.e., DLK1-DMR, IG-DMR and MEG3-DMR^14–17^), we investigated whether FAC could epigenetically alter the methylation and acetylation status in genomic regions potentially involved in miRNA regulation. Inhibition of methyltransferases and histone deacetylases using 5-Azacytidine (AZA) and Suberoylanilide Hydroxamic Acid (SAHA), respectively, resulted in rescuing the expression of miR-432-5p, miR-127-3p, with minimal effects on miR-138-5p expression. IPA analyses identified notable proteomic targets of key miRNAs including ALDH1A2 (for miR-138-5p target) and PAX8 (for miR-432-5p, miR-127-3p, and miR-138-5p). Although these targets could not be validated experimentally, TERT RNA was identified to be partially regulated by miR-138-5p. From a functional perspective, overexpression of these miRNAs reversed cell survival induced by chronic iron exposure in FT194 cells.

## Materials and methods

### Experimental design and statistical rationale

Understanding the mechanism underlying transformation of FTSECs requires the use of an in vitro model system, such as immortalized FT194 cells, generated via SV40 LTAg and hTERT stable expression, characterized by p53 inactivation, as described previously^18^. We previously reported the generation of oncogenically transformed FT194 cells; briefly, these were produced by stable overexpression of c-Myc^T58A^, H-Ras^V12A^, and SV40 LTAg^10^. In addition, we described the generation of a transformed-like FTSEC cell line following chronic iron overload (with 250 nM Ferric Ammonium Citrate (FAC) for > 60 days in culture) which was characterized by alterations in oncogene expression (based on a focused approach) and survivability^10^. Briefly, the FTSECs were seeded at 500 cells/well in 6 well plates and subsequently treated with a range of FAC doses (0, 25 nM, 250 nM, 2.5 µM, 25 µM, or 250 µM), as previously described^10^. Cell growth was continually monitored and cultures propagated in FAC-containing media (with media replenishments every 4 days). Cells treated with 250 nM FAC elicited greater cell numbers (compared with Untreated cells or other iron doses) and was therefore selected for expansion and experimentation along with Untreated control cells which were maintained concurrently. Our prior attempts with mM FAC doses similar to those previously reported^19,20^ was highly toxic and therefore not further pursued.

To further characterize global changes in an unbiased manner, we prepared experimental samples from Untreated (UNT), FAC-treated (FAC), control virus (CV), and oncogenic cocktail virus (OCV) infected FT194 cells; these were utilized for both noncoding RNA microarray and proteomic analyses. Specifically, the proteomic study utilized flash frozen cell pellets (UNT/FAC or CV/OCV) of ~ 500,000 cells per replicate, based on the protein extraction yield obtained by the S-trap sample processing approach previously reported by us^21,22^. For LFQ-based quantitation of protein expression, 5 replicates (from the same “batch” of UNT/FAC or CV/OCV cells) per group were utilized based on the expected quantitation precision of our approach obtained for cell lines^22^. For a statistical power of 90% with alpha = 0.05 and n = 5 replicates per group, an effect size of 2.348 would be needed based on using a two-tailed, unpaired t-test. The average coefficient of variation for each group was calculated for LFQ intensities obtained experimentally and then used to determine the fold change to achieve this effect size, which was then compared to the z-score cutoff used in this study (|z-score|> 1). A conventional FDR correction approach (e.g., Benjamini-Hochberg) was not employed given the tendency for decreased sensitivity; however, a combined filtering approach that considers variance and fold change was used (Welch’s t-test *p* < 0.05 and |z-score|> 1), which has been shown to adequately control FDR while maintaining sensitivity^23^.

For the miRNA analyses, total RNA was isolated from 3 replicates (from the same “batch” of UNT/FAC or CV/OCV cells), quantified by Nanodrop, and then analyzed using the GeneChip 4.1 Array (#902409, ThermoFisher Scientific, Waltham, MA, USA). The miRNA array contained 30,424 mature miRNAs, of which 2,578 were of human origin. To obtain a statistically relevant dataset of differentially expressed miRNA targets, an approach was applied of a > twofold change cutoff along with a FDR-adjusted *p* value < 0.05 top miRNA targets were selected to generate volcano plots and Venn diagrams.

### Cell culture and treatments

Human immortalized FTSECs (FT194) were provided by Dr. Ronald Drapkin (Department of Obstetrics and Gynecology, University of Pennsylvania, Philadelphia, PA, USA)^18^. These cells were immortalized by SV40 LTAg and hTERT, and were maintained in DMEM:F12 (1:1, #15-090-CV, Corning Incorporated, Corning, NY, USA) with phenol red, supplemented with 2% Ultroser G Serum Substitute (#67042, Crescent Chemical Company, Islandia, NY, USA) and 1% penicillin–streptomycin, as previously described^10^. Long-term FAC treated (annotated FAC or F) and the corresponding Untreated FT194 (annotated UNT or U) cells were maintained in phenol red-free DMEM:F12 (1:1, #21041–025, ThermoFisher, Waltham, MA, USA) with 8% charcoal dextran-stripped FBS and 1% penicillin/streptomycin (denoted as -PR media), as previously described^10^. Cells were incubated at 37 °C in a 5% CO_2_ environment. Cell lines were tested for mycoplasma and confirmed to be negative. Chronic iron-treated (250 nM for greater than 60 days) immortalized FT194 cells were maintained in 250 nM ferric ammonium citrate (FAC) (day 111 to 170 and *p* = 30–52)^10^. Oncogenic cocktail virus infected (OCV) and control virus infected FT194 cells (CV) cells generated by retroviral transduction of c-Myc^T58A^, H-Ras^V12A^, and SV40 LTAg cDNAs were generated previously^10^ and were used herein at passages of RV + 11.

FAC (#I72-500, Fisher Scientific, Pittsburgh, PA, USA) stock was prepared in PBS and used at a final concentration of 250 nM^10^. Stocks for the DNMT1 inhibitor, 5- Azacytidine (AZA, #S1782, Selleck Chemicals, Houston, TX) and the HDAC inhibitor, SAHA (#S1047, Selleck Chemicals, Houston, TX) were prepared in dimethylsulfoxide (DMSO). Both drugs were utilized at 0.5 µM, 1 µM, 5 µM, 10 µM, 25 µM and 50 µM final concentrations, which were based on a literature review of the most appropriate doses^24–27^. A dose range of 0.5 µM to 10 µM of AZA and 0.5 µM to 50 µM of SAHA (as mentioned previously^24–27^) were initially tested individually for 24 h in both Untreated and FAC-exposed FT194 cells (results not shown); the optimal dose was then selected for further experiments. Following optimization studies, 1 µM AZA and 50 µM SAHA doses were selected for use.

### Mass spectrometry-based proteomic analyses

Suspension trap (S-trap) sample processing of each experimental group (U, F, CV, OCV) was performed as previously described^22^ using an approximate 500,000 cell count for each group (n = 5 per group). Tryptic peptide concentrations were normalized based on the original protein concentration measurements determined by a Pierce 600 nm protein assay (Thermo Fisher Scientific). LC–MS/MS analysis of the cell lysate digests was performed using a hybrid quadrupole-Orbitrap instrument (Q Exactive Plus, Thermo Fisher Scientific) coupled to an Ultimate 3000 UPLC system (Thermo Fisher Scientific). Digested samples were first concentrated on a 2 cm × 75 µm ID PepMap C18 trap column (Thermo Fisher Scientific) followed by separation on a 55 °C-heated, 75 cm × 75 µm ID C18 PepMap column (Thermo Fisher Scientific). A 120 min gradient from 2 to 28% B, where B was 0.1% formic acid in 80% acetonitrile:20% water was used to separate peptides, as described in our prior publication^21^. An additional ramp to 40% B over 15 min followed by a wash at 95% B was implemented. For mass spectrometric analysis, data-dependent acquisition (DDA) with a top-10 method was utilized. The full MS spectra were acquired in the m/z range of 375–1200 at 70,000 resolution followed by MS/MS scans at 17,500 resolution. AGC target counts of 1E6 and 5E4 with maximum IT values of 20 and 50 ms for MS1 and MS2 were utilized, respectively. A normalized collision energy of 28 and isolation window of 1.6 m/z was employed with charge state exclusion set for unassigned, 1, 6–8, and > 8. Dynamic exclusion was set for 20 s with isotope exclusion enabled and the peptide match setting to preferred. All details of the mass spectrometry data acquisition and LC parameters are embedded in the raw data files, which have been deposited to the ProteomeXchange Consortium via the PRIDE^28^ partner repository with the dataset identifier PXD018416.

MaxQuant (version 1.6.6.0) was used to search raw files against the Uniprot protein database for *Homo sapiens* (version UP000005640, 71607 entries). Search parameters included the variable modifications of N-terminal protein acetylation and methionine oxidation as well as the constant modification of cysteine by carbamidomethylation. An additional database of known contaminants provided with MaxQuant was utilized where the first search tolerance was set to 20 ppm followed by a main search tolerance of 4.5 ppm, as described in our earlier work^21,22^. Furthermore, a search strategy using reversed sequences in a decoy database was employed to achieve protein and peptide FDR values of less than 1%^21,22^. Label free quantification (LFQ)-based quantitation was enabled, with a minimum ratio count of 1, and the “match-between-runs” feature using default settings was employed to increase proteomic identification, as described in our earlier work^21,22^.

The resulting Protein-Groups text file generated by MaxQuant was edited by removing the reverse and contaminant sequences as well as proteins only identified by modification (similarly described in our earlier work)^21^. The file was then uploaded into Perseus (version 1.6.1.1)^21^ twice for separate analysis of FAC-treated FT194 cells (F) relative to Untreated FT194 cells (U), and oncogenic cocktail virus infected FT194 cells (OCV) relative to control virus infected cells (CV). Each file was then analyzed whereby LFQ values were log_2_-transformed and proteins were removed that had missing values in more than just 2 out of the 5 replicates, similarly described in our earlier work^21^. The imputation function was utilized where missing values were replaced using width parameters of 0.3 for both and downshift parameters set to 1.8 and 1.75 for F vs. U and OCV vs. CV, respectively^21^. The average ratio of treatment over control was then calculated in Excel along with a Welch’s t-test (p-value < 0.05) and z-score (z-value > 1), similarly described in our earlier work^21^. These filtered lists containing protein identification and average ratio of each comparison were then uploaded to Ingenuity Pathway Analysis (IPA) in order to determine upstream regulator overlap and activity, over-represented canonical pathways, as well as other biological and disease functions (*p* < 0.05, Fisher’s exact test), similarly described in our earlier work^21^. Additionally, differentially expressed miRNAs (described below) were uploaded into IPA and paired against the proteins identified from proteomic analysis, which are known (experimentally determined) or predicted (moderate or high confidence) downstream targets of the miRNAs, through the miRNA Target Filter function. Paired miRNA-protein targets were filtered to include those in which the observed miRNA up- or down-regulation resulted in down- or up-regulation of the protein target, respectively. The corresponding network was reconstructed in IPA to demonstrate the potential regulatory role of each selected miRNA on the protein expression profile obtained.

### MicroRNA microarray

**Figure 1:**
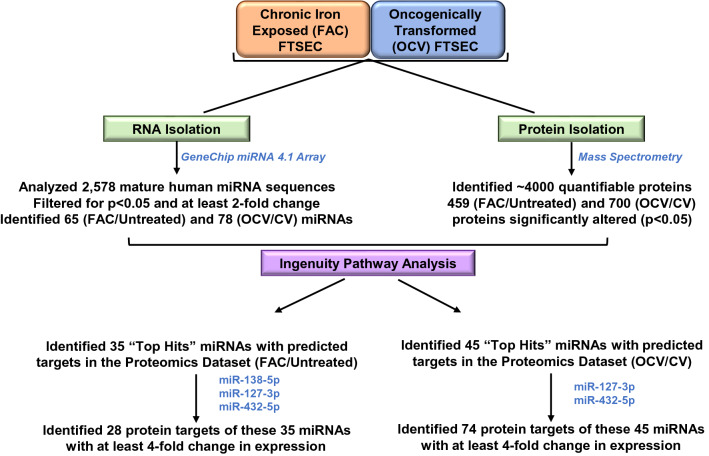
Schematic representation of the proteomics and microarray experimental strategy and analyses in FTSECs. Global proteomics analyses via mass spectrometry and miRNA analysis via Gene Chip miRNA 4.1 array were performed using chronic iron treated FT194 cells (*p* = 30 at day 104 of FAC treatment) and transformed FT194 cells (*p* = RV + 11) (with their corresponding control cells). This was followed by implementation of IPA to identify “top hits” of altered miRNA associated with protein changes.

Total RNA was isolated from FT194 cells maintained in 250 nM FAC for 104 days (p = 31) along with parental untreated FT194 cell line cultured simultaneously. In addition, total RNA was isolated from CV and OCV-infected FT194 cell lines (p = RV + 12). RNA isolation was performed using the RNeasy Kit (#74106, QIAGEN, Valencia, CA, USA) according to manufacturer’s instructions. Total RNA was quantified by Nanodrop and then analyzed using the GeneChip 4.1 Array (#902409, ThermoFisher Scientific, Waltham, MA, USA). The miRNA array contained 30,424 mature miRNAs, of which 2,578 were of human origin. To obtain a statistically relevant dataset of differentially expressed miRNA targets, an approach was applied of a > twofold change cutoff along with a non-adjusted *p*-value < 0.05; top miRNA targets were selected to generate volcano plots and Venn diagrams. miRNA array and proteomics data were combined in Ingenuity Pathway Analysis (IPA) to associate miRNAs with top proteomic hits. Thirty-five miRNAs had 28 protein targets identified in the proteomic screen in FAC-treated (compared to Untreated) FT194 cell samples whereas 45 miRNAs had 74 protein targets identified in the proteomic screen in transformed OCV (compared to control virus transfected), both with a cutoff of > fourfold change to focus on the “top hits” of biological relevance. The experimental strategy is depicted in Fig. 1.

### MicroRNA transfection

Untreated and FAC-treated FT194 cells were seeded at 250,000 cells (for protein isolation) in 6-well plates and 500,000 cells (for miRNA isolation) in 60 mm dishes. After overnight cell adherence, cells were transfected with 200 pmol control mimic (mirVana miRNA mimic Negative control 1, #4464059, Life Technologies, Grand Island, NY) or hsa-miR-138-5p (mirVana miRNA mimic, Assay ID# MC11727, Life Technologies, Grand Island, NY), hsa-miR-432-5p (mirVana miRNA mimic, Assay ID# MC10941, Life Technologies, Grand Island, NY), or hsa-miR-127-3p (mirVana miRNA mimic, Assay ID# MH10400, Life Technologies, Grand Island, NY) using Fugene HD (Promega, Madison, MI). Twenty-four hours post-transfection, cells were recovered and at 48 h post-transfection, protein lysates, total RNA, or miRNA were then collected.

### MicroRNA isolation for real-time PCR

miRNA was isolated using the *mir*Vana Isolation Kit (#AM1561, ThermoFisher Scientific, Waltham, MA, USA) following the manufacturer’s protocol. miRNA was quantified by Nanodrop and real-time PCR was performed using the TaqMan RNA-to-CT 1-Step Kit (#4392938, ThermoFisher Scientific, Waltham, MA, USA) with the following primer/probe sets: miR-432-5p (assay ID #: 001026), miR-127-3p (assay ID #: 000452), and miR-138-5p (assay ID #: 002284). The fold-change in miRNA expression was calculated using the 2^−ΔΔCT^ correlative method, in which C_T_ values were normalized to the RNU6B control (assay ID #: 001093, ThermoFisher Scientific, Waltham, MA, USA).

### Protein isolation, SDS-PAGE, and western blotting

Cell lysates were collected for separation on appropriate percentage SDS-PAGE gels and protein expression analyzed via western blotting using previously published methods^29,30^. Western blotting was performed using the following Cell Signaling Technology (Danvers, MA, USA) primary antibodies: DNMT1 rabbit monoclonal (1:1000, #5032), EVI1 rabbit monoclonal (1:500, #2593), Pan-Actin rabbit polyclonal (1:1000, #4968), Acetyl Histone H3 (Lys9/Lys14) rabbit polyclonal (1:1000, #9677), ALDH1A2 rabbit polyclonal (1:1000, #83805), and CRYAB rabbit monoclonal (1:1000, #45844). ITGA2 rabbit monoclonal (1:1000, MA535243) was obtained from ThermoFisher Scientific (Waltham, MA, USA). PAX8 rabbit polyclonal antibody (1:1000, #10336-1-AP) was obtained from Proteintech (Rosemont, IL, USA).

The western blots were developed at multiple exposures onto film, which were all scanned using a Flatbed Scanner (HP Scanjet 5590) and inserted as images into Powerpoint, without any manipulation (no contrast alterations) apart from cropping to within 6 band widths above and below the band of interest. Each developed blot (representing a specific antibody application) are presented in one powerpoint slide with space in-between, delineating different antibody applications to the same blot. The EVI1 and Pan-Actin antibodies were previously optimized in our laboratory^31^. The use of DNMT1^32^, Acetyl Histone H3^33^, and PAX8^34^ were utilized based on prior publications and ALDH1A2 was utilized based on data available by Cell Signaling Technology.

### EVI1 siRNA in FT194 cells

siRNA transfection in FT194 cells was performed as previously reported^10^. Briefly, cells were seeded in six-well plates at a density of 500,000 cells/well or at 1,000,000 cells/dish in 60 mm dishes followed by overnight adherence. ON-Target Plus non-targeting control siRNA (#D-001810-10-20, Dharmacon (Lafayette, CO, USA)) or EVI1-targeting siRNA (siB, custom designed as described previously)^10,31,35^ were transfected into cells using RNAiMax (#13778-075, Invitrogen, Carlsbad, CA, USA). Cells were recovered 24 h post transfection; cell lysates and miRNAs were collected at 48 h post-transfection for western blotting and real-time PCR, respectively.

### Bioinformatics of EVI1 binding site in miR-138-5p-1 promoter region

The UCSC Genome Browser (www.genome.ucsc.edu, Human Dec. 2013 (GRCh38/hg38 Assembly) was utilized to obtain the genomic sequence (5000 bp upstream) of the promoter region for miR-138-5p-1 (located at 3p21.32) and for miR-138-5p-2 (located at 16q13); miRBase was utilized to obtain the pre-miRNA sequence for these miRNAs. Previous research identified the DNA sequences that EVI1 binds to; EVI1 N-terminus binds to the sequence GACAAGATA^36,37^ while the C-terminus binds to the sequence GAAGATGAG^38,39^. Overall, the consensus EVI1 binding sequence is TGACAAGATAA^36,39^. Thus, these reported EVI1 binding sequences were aligned with the promoter regions for both miR-138-5p-1 and miR-138-5p-2 using the Genomatix software suite (version 3.11, http://www.genomatix.de/cgi-bin/dialign/dialign.pl).

### Statistical analyses

Data from real-time PCR, densitometry, and cell counting studies were analyzed using the Graphpad Prism software, version 6.04 (La Jolla, CA, USA). Error bars represent the mean ± SD and *p*-values were determined through the non-parametric Student’s *t*-test for which “ns” represents non-significant values, * indicates *p* ≤ 0.05, ** indicates *p* ≤ 0.01, *** indicates *p* ≤ 0.001, and **** indicates *p* ≤ 0.0001. Fold changes and percent reductions were calculated from the average of at least three independent experiments.

## Results

### miRNA and proteomic profiling of chronic FAC-treated and oncogenically transformed FTSECs

Elucidating molecular mechanisms involved in initiation and progression of ovarian cancer is essential towards developing novel therapeutic strategies. We have previously identified a subset of genes (including EVI1, located at 3q26.2 in HGSOC) that were altered following long-term iron exposure in FTSECs^10^. However, to acquire a comprehensive understanding of the iron-induced alterations, a global mass spectrometry-based proteomics analysis was performed in FAC-exposed and Untreated FT194 cells. We then compared the resultant proteomic alterations to OCV- and CV-infected FT194 cells, as generated previously^10^. For FAC-exposed FT194 cells compared to Untreated cells, 4402 total proteins were identified with 3968 quantifiable proteins after filtering. In OCV-infected compared to CV-infected FT194 cells, 4,691 total proteins were identified with 4148 quantifiable proteins after filtering. The average coefficient of variation for the LFQ values of CV-infected, OCV-infected, FAC-treated, and Untreated FT194 cells was 25.4, 21.4, 21.9, and 19.6%, respectively. The median coefficient of variation for the LFQ values of CV-infected, OCV-infected, FAC-treated, and Untreated FT194 cells was 16.8, 13.7, 15.0, and 11.7%, respectively. Statistically significant “top hits” were obtained using Welch’s t-test (*p* < 0.05) and z-score (z-score > 1) which identified 700 protein targets in the OCV (relative to CV) cells and 459 protein targets in iron-exposed FTSECs (relative to Untreated). To achieve the effect size corresponding to a statistical power of 0.9 based on our experimental conditions, a fold-change of ~ 1.7–1.8 would be needed (assuming the global average of the coefficient of variation determined for each group), which is consistent with the implemented z-score cutoff. Additional restrictions were applied to this data set to identify the protein targets that had LFQ intensity ratio of ≥ 2 or ≤ 0.5. Using this strategy, we thus identified 622 targets for OCV (relative to CV) and 243 targets for FAC-exposed FTSECs (relative to Untreated) (Fig. 1). The list of total quantifiable as well as differentially expressed proteins are provided as supplemental Tables 1 and 2.

**Figure 2:**
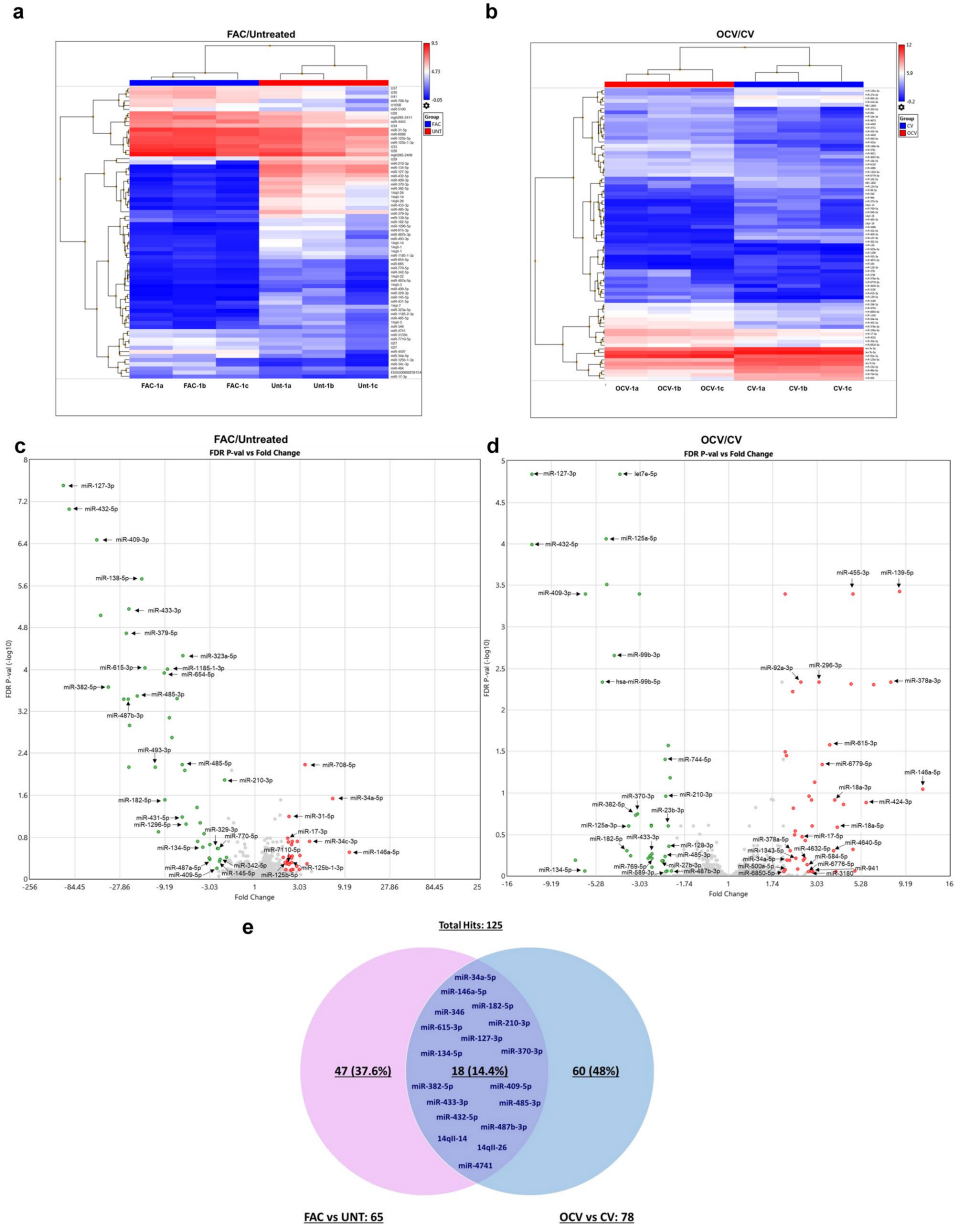
Altered miRNAs identified from microarray analysis in FTSECs. Heat Map representing miRNAs derived from the microarray analysis showing a decrease (Blue) and increase (Red) in miRNAs with (**a**) FAC treatment (FAC-1a, 1b, 1c) compared to Untreated and (**b**) transformed OCV- relative to CV-infected FT194 cells. Dysregulated miRNAs are displayed as Volcano Plots in (**c**) FAC-treated vs Untreated FT194 cells, (**d**) Transformed (OCV/CV) and, (**e**) dysregulated miRNAs are displayed in a Venn diagram showing 18 common (overlapping) miRNAs between FAC-treated relative to Untreated as well as transformed OCV relative to CV analyses.

Since alterations in miRNAs can contribute to cancer pathogenesis by targeting multiple protein targets^40,41^, we performed microRNA array profiling using the GeneChip miRNA 4.1 Array. We compared chronic FAC-exposed FTSECs relative to Untreated cells as well as OCV- relative to CV-infected FTSECs, which identified a total of 65 and 78 unique non-coding RNAs, respectively, including mature miRNAs and snoRNAs (7 snoRNAs were repeated as duplicates in the dataset resulting in a total of 71 and 79 ncRNAs identified in FAC/UNT and OCV/CV analyses, respectively), with at least a twofold change (*p* value > 0.05, displayed as a heat map, volcano plot, and Venn diagram (Fig. 2a–e). There were 42 upregulated, 29 downregulated miRNAs in FAC-treated (relative to Untreated) and 45 upregulated, 34 downregulated miRNAs in OCV (relative to CV). miRNAs downregulated in FAC-treated (relative to Untreated) cells showed higher fold change (X-axis) and statistical significance (p-value at Y-axis) compared to OCV cells (relative to CV) (Fig. 2c, d*)*. Since the quantity of changes are more numerous in the OCV versus CV FTSEC comparison (relative to FAC versus Untreated FTSEC), this suggests that the combination of p53 inactivation, c-Myc^T58A^ expression, and H-Ras^V12A^ expression (within the OCV cocktail) may mediate increased neoplastic cellular alterations.

**Figure 3:**
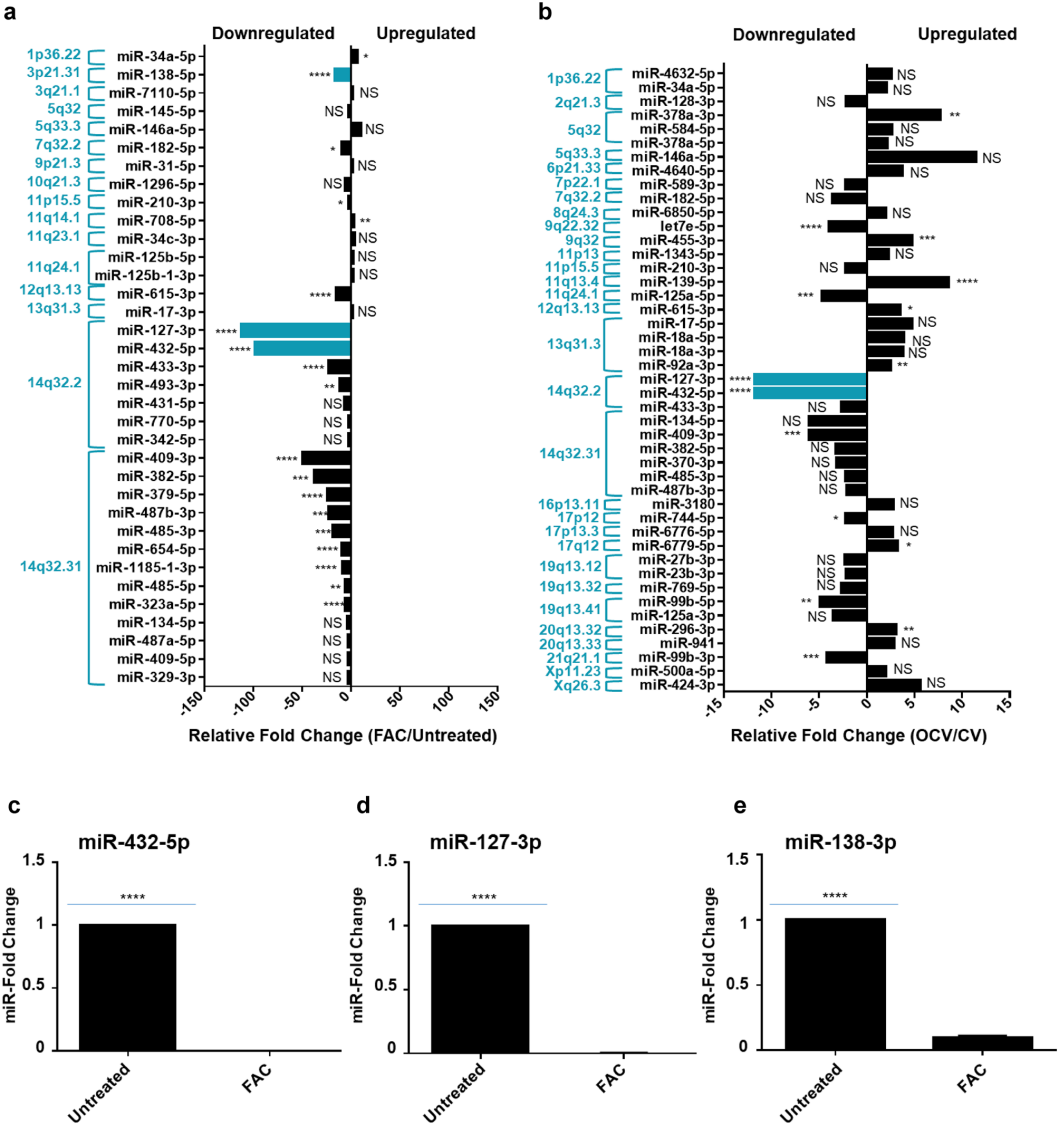
Top miRNAs altered in FT194 cells and their genomic locations. These graphs show the miRNA targets (analyzed via microarray profiling) which correlate with the protein changes (analyzed via mass spec proteomics analysis) as predicted by IPA. The relative fold change of miRNAs is represented and organized according to their genomic locations in (**a**) 250 nM FAC-treated FT194 cells compared to Untreated, and (**b**) FT194 transformed cells via Oncogenic cocktail virus (OCV), compared to control virus (CV) cells. Real-time PCR analysis of (**c**) miR-432-5p, (**d**) miR-127-3p, and (**e**) miR-138-5p was performed after isolating total miRNAs from 250 nM FAC-treated cells as compared to the Untreated, at days 111 and 124 of FAC treatment (*p* = 33 and 37), to validate the downregulation of these miRNAs as predicted by IPA analysis. RNU6B was used as a reference control and the data represents composite of three independent experiments.

Since alterations in protein expression can be regulated post-transcriptionally by miRNAs, we integrated the top miRNA hits with the protein targets using IPA to identify associations between the two analyses. Integrated proteomics and microarray analyses identified 35 miRNAs in FAC-treated FT194 cells (relative to Untreated) and 45 miRNAs in OCV cells (relative to CV cells) with the following characteristics: (1) > twofold change and (2) with direct protein targets that were altered with respect to miRNA levels (Fig. 3a, b). Twenty of the 35 miRNAs (57.14%) altered in FAC-treated cells (relative to Untreated) were located at chromosome 14q32 (Fig. 3a), while only 9 out of the 45 miRNAs (20%) altered in OCV cells (relative to CV) were located at this region (Fig. 3b). This region, amongst others, also harbors common fragile sites (Tables 1 and 2), possibly rendering the chromosomal loci susceptible to replication stress, which is known to impact genomic stability in many cancers^42^. Interestingly, the 14q32 locus contains a cluster of 54 miRNAs, one of the largest miRNA clusters in the human genome^43^. Many of these miRNAs appear to be downregulated in multiple cancer types associated with tumor suppressive properties^11,27,44–48^ and oncogenic properties^49^. From the miRNA profiling, we identified that two miRNAs from this cluster, miR-432-5p and miR-127-3p, were 97.9- and 111.7-fold downregulated, respectively, in FAC-treated cells in contrast to only 11.7-fold downregulated in OCV cells. These results were validated by real-time PCR in FAC-treated FT194 cells (Fig. 3c, d), which showed a 99.9% reduction for miR-432-5p and miR-127-3p (*p* value < 0.0001) in FAC-treated relative to Untreated FT194 cells. Additionally, among other highly dysregulated miRNAs from other genomic regions such as 3p21.31, we identified that miRNA-138-5p (located at 3p21) was 16.3-fold downregulated with chronic FAC-exposure in FT194 cells (Fig. 3a) and validated via qPCR to be 90.1% reduced (*p* value < 0.0001, Fig. 3e). miR-138-5p also appears to be commonly downregulated in multiple cancers^50–53^.

**Table 1:** The common fragile sites associated with the top 35 miRNA hits in FAC-exposed relative to Untreated FT194 cells are shown in this table, with > twofold change cutoff along with a FDR-adjusted *p* value < 0.05.

*FAC vs. Untreated*					
miRNA ID	Genomic location	Fold Change	FDR *p* value		Nearest common fragile site
miR-34a-5p	1p36.22	6.85	0.02890000	*	FRA1A (1p36)
miR-138-5p	3p21.31	−16.25	0.00000189	****	FRA3A (3p24.2); FRA3B (3p14.2)
hsa-miR-7110-5p	3q21.1	2.29	0.49900000	NS	FRA3D (3q25)
miR-145-5p	5q32	−2.38	0.45400000	NS	FRA5C (5q31.1)
miR-146a-5p	5q33.3	10.26	0.31070000	NS	FRA5C (5q31.1)
mir-182-5p	7q32.2	−9.19	0.03020000	*	FRA7G (7q31.2); FRA7H (7q32.3
miR-31-5p	9p21.3	2.32	0.06330000	NS	N/A
miR-1296-5p	10q21.3	−5.42	0.08800000	NS	FRA10D (10q22.1)
miR-210-3p	11p15.5	−2.09	0.01260000	*	FRA11C (11p15.1)
miR-708-5p	11q14.1	3.44	0.00660000	**	FRA11F (11q14.2)
miR-34c-3p	11q23.1	3.83	0.19130000	NS	FRA11G (11q23.3)
miR-125b-5p	11q24.1	2.45	0.48070000	NS	FRA11G (11q23.3)
miR-125b-1-3p	11q24.1	2.45	0.48070000	NS	FRA11G (11q23.3)
miR-615-3p	12q13.13	−15.09	0.00009200	****	N/A
miR-17-3p	13q31.3	2.24	0.16410000	NS	FRA13D (13q32)
miR-127-3p	14q32.2	−111.69	0.00000003	****	FRA14C (14q24.1)
miR-432-5p	14q32.2	−97.87	0.00000009	****	FRA14C (14q24.1)
miR-433-3p	14q32.2	−22.41	0.00000703	****	FRA14C (14q24.1)
miR-493-3p	14q32.2	−11.59	0.00730000	**	FRA14C (14q24.1)
miR-431-5p	14q32.2	−6.02	0.06480000	NS	FRA14C (14q24.1)
hsa-miR-770-5p	14q32.2	−2.45	0.25680000	NS	FRA14C (14q24.1)
miR-342-5p	14q32.2	−2.34	0.41920000	NS	FRA14C (14q24.1)
miR-409-3p	14q32.31	−49.16	0.00000034	****	FRA14C (14q24.1)
miR-382-5p	14q32.31	−37.07	0.00020000	***	FRA14C (14q24.1)
miR-379-5p	14q32.31	−23.84	0.00002020	****	FRA14C (14q24.1)
miR-487b-3p	14q32.31	−22.55	0.00040000	***	FRA14C (14q24.1)
miR-485-3p	14q32.31	−18.02	0.00030000	***	FRA14C (14q24.1)
hsa-miR-654-5p	14q32.31	−9.37	0.00010000	****	FRA14C (14q24.1)
hsa-miR-1185–1-3p	14q32.31	−8.64	0.00009730	****	FRA14C (14q24.1)
miR-485-5p	14q32.31	−5.98	0.00660000	**	FRA14C (14q24.1)
miR-323a-5p	14q32.31	−5.85	0.00005460	****	FRA14C (14q24.1)
miR-134-5p	14q32.31	−3.58	0.24840000	NS	FRA14C (14q24.1)
miR-487a-5p	14q32.31	−3.04	0.41920000	NS	FRA14C (14q24.1)
miR-409-5p	14q32.31	−2.54	0.61360000	NS	FRA14C (14q24.1)
miR-329-3p	14q32.31	−2.52	0.25680000	NS	FRA14C (14q24.1)

****p < 0.0001.

***p < 0.001.

**p < 0.01.

*p < 0.05.

NS *p* > 0.05.

**Table 2:** The common fragile sites associated with the top 45 miRNA hits in OCV relative to CV FT194 cells are shown in this table, with > twofold change cutoff along with a FDR-adjusted *p*-value < 0.05.

*OCV vs. CV*					
miRNA ID	Genomic location	Fold change	FDR *p* value		Nearest common fragile site
hsa-miR-4632-5p	1p36.22	2.6	0.61160000	NS	FRA1A (1p36)
miR-34a-5p	1p36.22	2.08	0.64000000	NS	FRA1A (1p36)
miR-128-3p	2q21.3	−2.11	0.43710000	NS	FRA2F (2q21.3)
miR-378a-3p	5q32	7.66	0.00460000	**	FRA5C (5q31.1)
miR-584-5p	5q32	2.67	0.73370000	NS	FRA5C (5q31.1)
miR-378a-5p	5q32	2.17	0.49590000	NS	FRA5C (5q31.1)
miR-146a-5p	5q33.3	11.43	0.08990000	NS	FRA5C (5q31.1)
miR-4640-5p	6p21.33	3.72	0.49590000	NS	FRA6C (6p22.2)
miR-589-3p	7p22.1	−2.2	0.88380000	NS	FRA7B (7p22)
miR-182-5p	7q32.2	−3.58	0.49590000	NS	FRA7G (7q31.2); FRA7H (7q32.3
miR-6850-5p	8q24.3	2	0.87770000	NS	FRA8D (8q24.3)
let7e-5p	9q22.32	−3.92	0.00001450	****	FRA9D (9q22.1)
miR-455-3p	9q32	4.77	0.00040000	***	FRA9E (9q32)
miR-1343-5p	11p13	2.31	0.61160000	NS	FRA11E (11p13)
miR-210-3p	11p15.5	−2.19	0.10890000	NS	FRA11C (11p15.1)
miR-139-5p	11q13.4	8.53	0.00040000	***	FRA11H (11q13)
miR-125a-5p	11q24.1	−4.65	0.00008670	****	FRA11G (11q23.3)
miR-615-3p	12q13.13	3.55	0.02630000	*	N/A
miR-17-5p	13q31.3	4.75	0.33370000	NS	FRA13D (13q32)
miR-18a-5p	13q31.3	3.9	0.25760000	NS	FRA13D (13q32)
miR-18a-3p	13q31.3	3.79	0.12120000	NS	FRA13D (13q32)
miR-92a-3p	13q31.3	2.48	0.00460000	**	FRA13D (13q32)
miR-127-3p	14q32.2	−11.76	0.00001450	****	FRA14C (14q24.1)
miR-432-5p	14q32.2	−11.75	0.00010000	****	FRA14C (14q24.1)
miR-433-3p	14q32.2	−2.62	0.53880000	NS	FRA14C (14q24.1)
miR-134-5p	14q32.31	−6.06	0.61160000	NS	FRA14C (14q24.1)
miR-409-3p	14q32.31	−6.04	0.00040000	***	FRA14C (14q24.1)
miR-382-5p	14q32.31	−3.22	0.18450000	NS	FRA14C (14q24.1)
miR-370-3p	14q32.31	−3.13	0.17770000	NS	FRA14C (14q24.1)
miR-485-3p	14q32.31	−2.22	0.58160000	NS	FRA14C (14q24.1)
miR-487b-3p	14q32.31	−2.04	0.87160000	NS	FRA14C (14q24.1)
miR-3180	16p13.11	2.82	0.87770000	NS	N/A
miR-744-5p	17p12	−2.21	0.03970000	*	N/A
miR-6776-5p	17p13.3	2.72	0.87770000	NS	N/A
hsa-miR-6779-5p	17q12	3.24	0.04560000	*	N/A
miR-27b-3p	19p13.12	−2.31	0.65520000	NS	N/A
miR-23b-3p	19p13.12	−2.14	0.24790000	NS	N/A
miR-769-5p	19q13.32	−2.62	0.61160000	NS	N/A
hsa-miR-99b-5p	19q13.41	−4.88	0.00460000	**	N/A
miR-125a-3p	19q13.41	−3.51	0.24790000	NS	N/A
miR-296-3p	20q13.32	3.11	0.00460000	**	N/A
miR-941	20q13.33	2.87	0.87720000	NS	N/A
miR-99b-3p	21q21.1	−4.18	0.00040000	***	N/A
miR-500a-5p	Xp11.23	2.03	0.84280000	NS	N/A
miR-424-3p	Xq26.3	5.6	0.13100000	NS	FRAXD (Xq27.2)

****p < 0.0001.

***p < 0.001.

**p < 0.01.

*p < 0.05.

NS *p* > 0.05.

**Figure 4:**
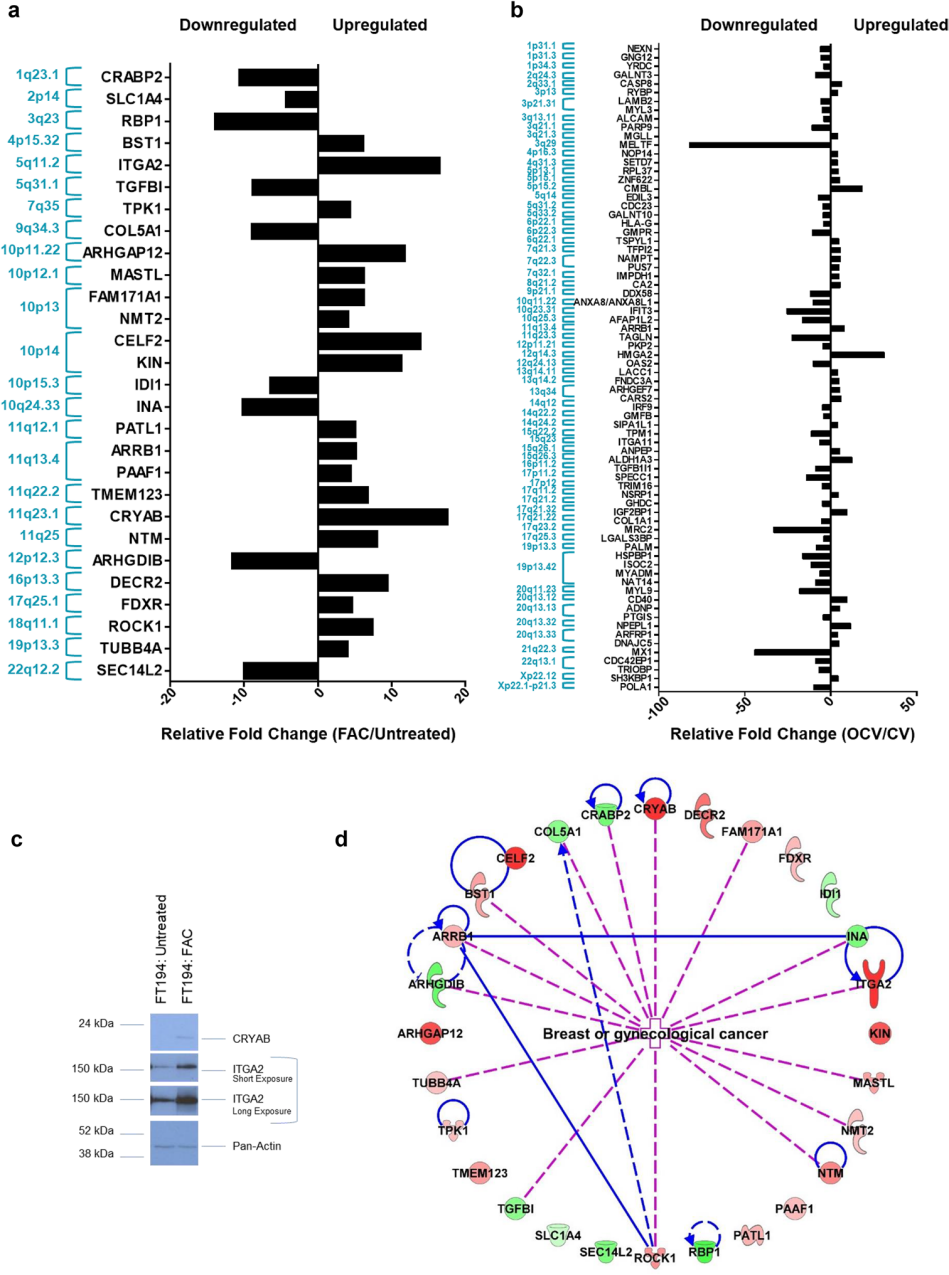
Protein targets and their genomic locations associated with top miRNAs altered in FT194 cells. These graphs show the protein targets associated with highly dysregulated miRNAs in FT194 cells, as determined by IPA analysis. Protein targets with > fourfold change, corresponding to the miRNAs, were compiled and organized by their genomic locations. Relative fold change of these protein targets has been represented for (**a**) 250 nM FAC-treated FT194 cells compared to Untreated, and (**b**) transformed OCV cells compared to CV cells. (**c**) Western Blotting analysis of 250 nM FAC-exposed and Untreated FT194 cells, using cell lysates collected at day 170 of FAC treatment (*p* = 52). Three independent replicates were performed; representative cropped blots are displayed. White space between cropped blots delineate different antibody applications to the same blot. The full-length uncropped blots are displayed in the Supplementary Information File. (**d**) Top 28 protein targets altered in gynecological cancers, with > fourfold change, identified for 250 nM FAC-treated FT194 cells compared to Untreated. Upregulated proteins are shown in red and downregulated proteins in green, ranging color intensity based on the fold change associated with each. The detailed legend for all molecular processes in the protein network is included in tabular form in supplementary Fig. S6.

We identified 28 protein targets in FAC-treated relative to Untreated cells and 74 protein targets in OCV relative to CV cells out of the total statistically significant “top hits” (*p* < 0.05 and z-value > 1), which were associated with characteristics of gynecological cancers (Fig. 4a, b). In Fig. 4c, we validated two of these “top hits”, namely CRYAB and ITGA2 via western blotting, which have roles as molecular chaperones^54^ and in adhesion to the extracellular matrix^55^, respectively.

The number of identified proteins and miRNAs from the OCV FT194 cells (relative to CV) were higher as compared to FAC-exposed cells (relative to Untreated), which suggests that the extent of neoplastic alterations induced by the oncogenic cocktail (hTERT, LTAg, c-Myc^T58A^, and H-Ras^V12A^) is more extensive than that induced by iron exposure (see Experimental Procedures). These ”hits” were not validated due to the expectation that oncogenic transformation of FTSECs would lead to a comparatively larger array of alterations recapitulating more closely tumorigenic profiles of ovarian tumors relative to chronic iron exposed FTSECs, which is the main focus of this study.

As the protein targets from the FAC versus Untreated analyses were found to be involved in multiple signaling and molecular pathways (Fig. 4d and Table 3), we further investigated the mechanism of FAC-induced dysregulation in FT194 cells, specifically focusing on analyzing the mechanism by which FAC alters miR-138-5p, miR-432-5p, and miR-127-3p levels to potentially contribute towards increased tumorigenesis.

**Table 3:** Top upregulated and downregulated protein targets associated with gynecological cancers identified via IPA in FAC-exposed relative to Untreated FT194 cells are shown in this table, with *p* < 0.05 and z-value > 1.

Protein target	Alias	Chromosomal location	Fold-change (Welch's T-test LFQ intensity F v U)	UniProtKB/Swiss-Prot summary
ARHGAP12	Rho GTPase Activating Protein 12	10p11.22	11.77340121	A member of a large family of proteins that activate Rho-type GTP metabolizing enzymes
ARHGDIB	Rho GDP Dissociation Inhibitor Beta	12p12.3	0.086813615	Regulates the GDP/GTP exchange reaction of the Rho proteins
ARRB1	Arrestin Beta 1	11q13.4	5.124634149	Functions in regulating agonist-mediated G-protein coupled receptor signaling by mediating both receptor desensitization and resensitization processes
BST1	Bone Marrow Stromal Cell Antigen 1	4p15.32	6.122146871	Synthesizes the second messengers cyclic ADP-ribose and nicotinate-adenine dinucleotide phosphoate
CELF2	CUGBP Elav-like Family Member 2	10p14	13.84767274	RNA-binding protein implicated in the regulation of several post-transcriptional events
COL5A1	Collagen Type V Alpha 1 Chain	9q34.3	0.112864701	Type V collagen is a member of group I collagen (fibrillar forming collagen)
CRABP2	Cellular Retinoic Acid Binding Protein 2	1q23.1	0.09458846	Transports retinoic acid to the nucleus
CRYAB	Crystallin Alpha B	11q23.1	17.53439428	May contribute to the transparency and refractive index of the lens
DECR2	2,4-Dienoyl-CoA Reductase 2	16p13.3	9.463406179	Participates in the degradation of unsaturated fatty enoyl-CoA esters having double bonds in both even- and odd-numbers positions in peroxisome
FAM171A1	Family With Sequence Similarity 171 Member A1	10p13	6.197873721	Involved in the regulation of the cytoskeletal dynamics/plays a role in actin stress fiber formation
FDXR	Ferredoxin Reductase	17q25.1	4.595277	Serves as the first electron transfer protein in all the mitochondrial P450 systems
IDI1	Isopentenyl-Diphosphate Delta Isomerase 1	10p15.3	0.156436427	Catalyzes the 1,3-allylic rearrangement of the homoallylic substrate isopentenyl to its highly eletrophilic allylic isomer, dimethylallyl diphosphate
INA	Internexin Neuronal Intermediate Filament Protein Alpha	10q24.33	7.346539312	Class-IV neuronal filament that is able to self-assemble and is involved in the morphogenesis of neurons
ITGA2	Integrin Subunit Alpha 2	5q11.2	16.44875121	Integrin alpha-2/beta-1 is a receptor for laminin, collagen, collagen C-propeptides, fibronectin, and E-cadherin
KIN	Kin17 DNA and RNA Binding Protein	10p14	11.27786487	Involved in DNA replication and the cellular response to DNA damage
MASTL	Microtubule Associated Serine/Threonine Kinase Like	10p12.1	6.20270082	Serine/threonine kinase that plays a key role in M phase by acting as a regulator of mitosis entry and maintenance'\
NMT2	N-myristoyltransferase 2	10p13	4.073197008	Adds a myristoyl group to the N-terkinal glycine residue of certain cellular and viral proteins
NTM	Neurotrimin	11q25	7.970555423	Neural cell adhesion molecule
PAAF1	Proteasomal ATPase Associated Factor 1	11q13.4	4.390010657	Inhibits proteasome 26S assembly and proteolytic activity by impairing the association of the 19S regulatiry complex with the 20S core
PATL1	PAT1 Homolog 1, Processing Body mRNA Decay Factor	11q12.1	5.037787567	RNA-binding protein involved in deadenylation-dependent decapping of mRNAs, leading to the degradation of mRNAs
RBP1	Retinol Binding Protein 1	3q23	0.072160245	Cytoplasmic retinol-binding protein
ROCK1	Rho Associated Coiled-Coil Containing Protein Kinase 1	18q11.1	7.346539312	Rho-kinases are serine/threonine kinases activated by RhoA GTPases
SEC14L2	SEC14 Like Lipid Binding 2	22q12.2	0.10067807	Carrier protein. Binds to some hydrophobic molecular and promotes their transfer between the different cellular sites
SLC1A4	Solute Carrier Family 1 Member 4	2p14	0.235645725	Transporter for alanine, serine, cysteine, and threonine
TGFBI	Transforming Growth Factor Beta 1	19q13.2	0.113836913	Multifunctional protein that regulates growth and differentation of various cell types and is invovled in various processes such as normal development, immune function, microglia function, and responses to neurodegeneration
TMEM123	Transmembrane Protein 123	11q22.2	6.71872719	Implicated in oncotic cell death, characterized by cell swelling, organelle swelling, vacuolization, and increased membrane permeability
TPK1	Thiamin Pyrophosphokinase 1	7q35	4.34870065	Catalyzes the phosphorylation of thiamine to thiamine pyrophosphate
TUBB4A	Tubulin Beta 4A Class IVa	19p13.3	4.021247785	Tubulin is the major constituent of microtubules

### FAC-induced epigenetic regulation of PAX8

**Figure 5:**
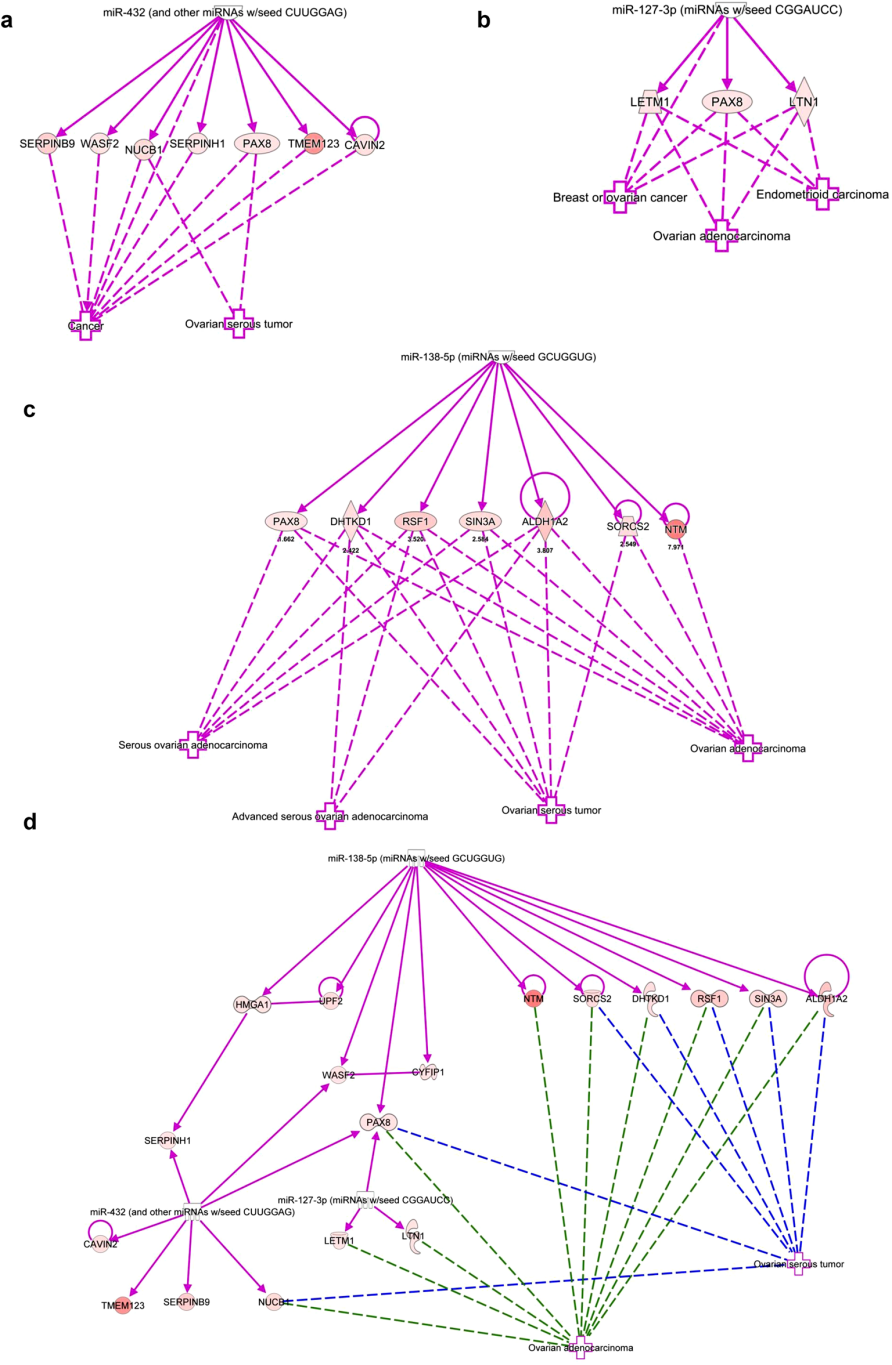
Protein targets of miR-138-5p, miR-432-5p, and miR-127-3p associated with gynecological cancers, predicted via IPA analysis. In co-relation with ovarian serous tumors, (**a**) 7 targets identified for miR-432 analysis and in correlation with gynecological malignancies, (**b**) 3 targets identified for miR-127-3p and (**c**) 7 targets for miR-138-5p analysis. (**d**) Combined target network analysis of miR-432, miR-127-3p and miR-138-5p in relation to their association with ovarian carcinoma revealed PAX8 as a common target of all three miRNAs.

**Figure 6:**
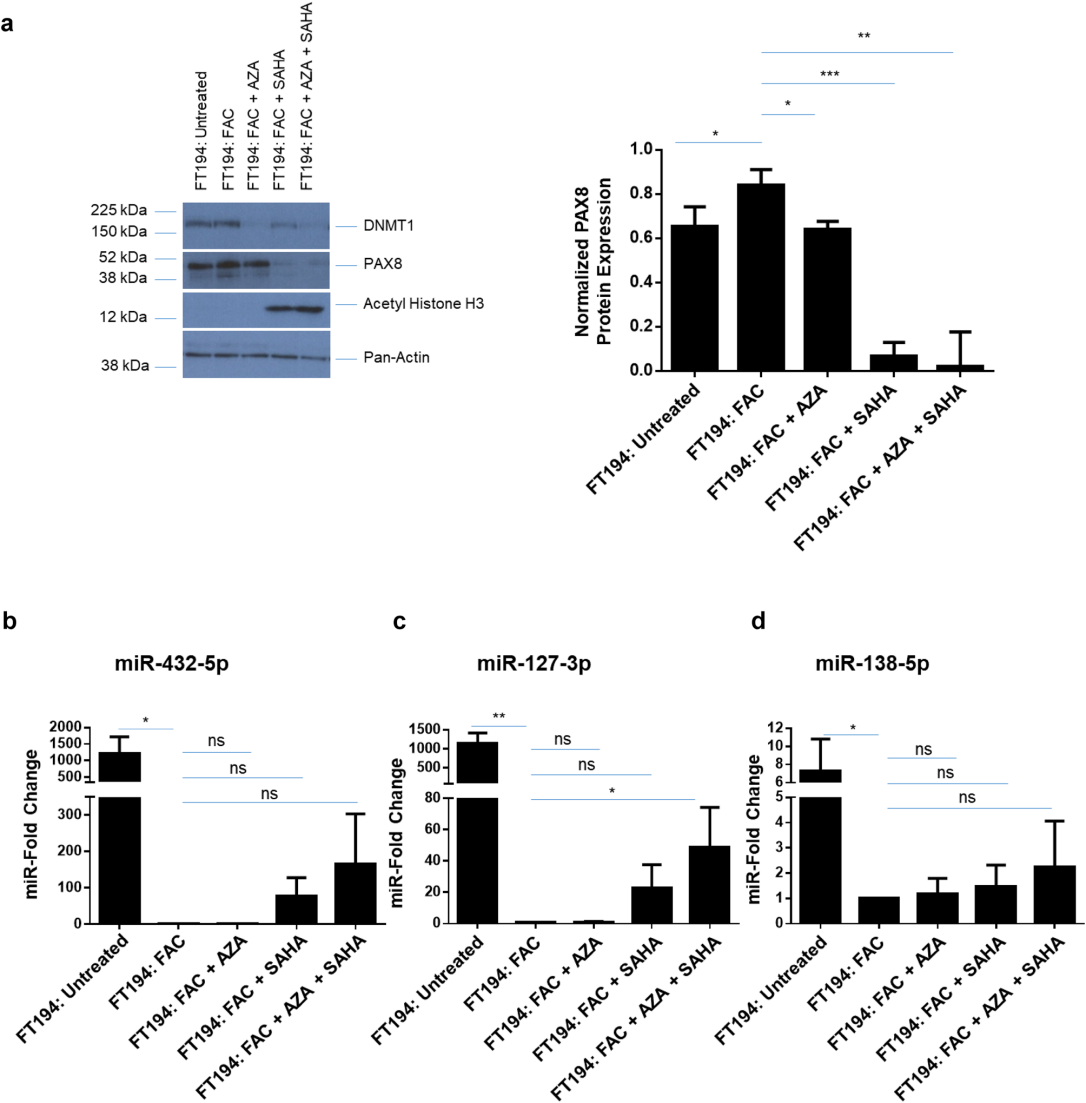
miR-432-5p, miR-127-3p, and miR-138-5p are epigenetically regulated by FAC. (**a**) Western Blotting analysis of 250 nM FAC-exposed FT194 cells treated with 1 µM AZA and/or 10 µM SAHA for 24 h, using cell lysates collected at days 129, 134 and 137 of FAC treatment (*p* = 38 and 39). Three independent replicates were performed; representative cropped blots and the densitometric analysis for PAX8 are displayed. White space between cropped blots delineate different antibody applications to the same blot. The full-length uncropped blots are displayed in the Supplementary Information File. Real-time PCR analyses of (**b**) miR-432-5p, (**c**) miR-127-3p, and (**d**) miR-138-5p in 250 nM FAC-exposed FT194 cells treated with 1 µM AZA and/or 10 µM SAHA for 24 h. The data shown represents the composite of three independent experiments. RNU6B was used as a reference control.

Integrated microarray and proteomics analyses were used to identify the most common protein targets of miR-432-5p, miR-127-3p, and miR-138-5p in FAC-treated cells relative to Untreated (Fig. 5a–c and supplementary Fig. S1) as well as in OCV (relative to CV) FT194 cells (supplementary Fig. S2–S4). Via IPA, the analyses identified PAX8 (Paired Box 8) as a potential common target of miR-432-5p, miR-127-3p, and miR-138-5p involved in ovarian cancer pathophysiology (Fig. 5d). Global proteomic analyses demonstrated that PAX8 was increased 1.7-fold in FAC-exposed FT194 cells, which was validated by western blotting and densitometric analyses (Fig. 6a, left and right panels). Since iron-overload conditions are associated with epigenetic changes in various human tissues^56,57^ and epigenetic modification of PAX8 is reported in ovarian cancer^58,59^, we next investigated whether FAC-induced increase of PAX8 could be altered with AZA and/or SAHA treatment. As shown in Fig. 6a, we observed a 95.6%-fold reduction in PAX8 protein following combinatorial treatment of SAHA and AZA. These results suggest that the increased PAX8 protein is epigenetically regulated as a result of chronic iron treatment in FT194 cells.

### FAC-induced epigenetic regulation of miRNAs at 14q32 and 3p21

miRNAs are also regulated by epigenetic mechanisms^60–62^ and miRNAs at chromosome 14q32 appear to be transcribed as a polycistronic miRNA cluster under control of epigenetic mechanisms^63,64^. In specific cancers, there is evidence to support hypermethylation at the 14q32 differentially methylated CpG regions (DMRs) which leads to cancer development^27,65^; furthermore, promoter hypoacetylation, which can recruit HDACs through methyl CpG binding proteins can also regulate gene expression at this locus^66^. In view of these regulatory mechanisms and the identification of twenty 14q32 miRNAs that were altered in FAC-exposed FT194 cells, we hypothesized that inhibition of DNA methylation with AZA and/or inhibition of histone deacetylases with SAHA may alter the expression of miR-432-5p and miR-127-3p (refer to Fig. 2 for “top hits” identified in the screening approach). Since miR-138-5p is associated with epigenetic changes^67,68^, we therefore investigated whether AZA and SAHA treatments could alter miR-138-5p levels. Inhibition of DNA methyltransferase with AZA was validated by western blotting for DNMT1, which showed reduced protein levels (Fig. 6a). In addition, HDAC inhibition was validated by demonstrating increased acetyl histone H3 levels via western blotting (Fig. 6a). Although SAHA treatment was more potent alone compared to AZA alone, the combination of AZA with SAHA, resulted in a further fold-increase of 164.8, 48.8 and 2.3 in miR-432-5p, miR-138-5p, and miR-127-3p levels, respectively, as measurement via real-time PCR (Fig. 6b–d). These results can be explained by the ability of the HDAC inhibitor, SAHA, to inhibit not only HDAC but also DNMT1 protein expression via inhibition of MAPK and thus the DNA methylation status^69^. HDAC inhibitors can also target DNMTs for degradation via the ubiquitin–proteasome-pathway by an Hsp90 chaperone mediated mechanism^70^. On the other hand, AZA specifically inhibits only DNMTs^71^.

### FAC-induced miR-138-5p downregulation is mediated independently of EVI1

**Figure 7:**
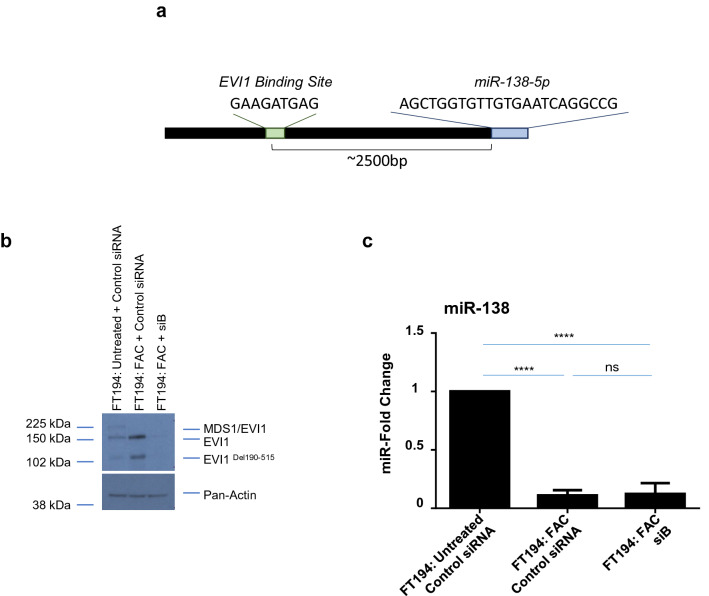
EVI1 knockdown does not alter miR-138-5p expression level. (**a**) Schematic representing the predicted EVI1 binding site upstream from miR-138-5p. EVI1 splice variants were reduced in FAC-treated FT194 cells using siRNA (siB) in FAC-treated cells. Cell lysates and miRNAs were collected at days 125 and 128 of FAC treatment (*p* = 37 and 38). (**b**) Western blotting was performed to validate the knockdown. Three independent experiments were completed and a representative blot is shown. (**c**) Real-time PCR of miR-138-5p was performed using RNU6B as a reference control. The data represents the composite of three independent experiments. White space between cropped blots delineate different antibody applications to the same blot. The full-length uncropped blots are displayed in the Supplementary Information File.

We have previously reported that telomerase reverse transcriptase (TERT) can be transcriptionally regulated by EVI1 (genomically amplified at chromosome 3q26) in FAC exposed FT194 cells^10^. Prior work has shown that miR-138 levels are inversely correlated with TERT as a result of direct binding of miR-138 to the 3′-UTR of TERT^72–74^. Therefore, we hypothesized that the EVI1 may upregulate TERT transcripts in a miR-138-dependent manner. Mature miR-138 originates from two primary transcripts: pri-miR-138-1 (encoded on chromosome 3p21, Genecard) and pri-miR-138–2 (encoded on chromosome 16q13, Genecard). Indeed, we identified predicted EVI1 binding sites ~ 2500 bp upstream of miR-138-5p-1 (Fig. 7a and supplementary Fig. S5); specifically, the C-terminal binding site, GAAGATGAG, was 100% aligned in this region, while the N-terminal and consensus sequences were imperfectly aligned (5 out of 9 nucleotides and 5 out of 11 nucleotides, respectively). There was also a potential binding site ~ 1000 bp upstream of miR-138-5p-2, but the binding sites were < 100% aligned, of which 8 out of 9 nucleotides aligned for the N-terminal binding site, 5 out of 9 nucleotides aligned for the C-terminal binding site, and 8 out of 11 nucleotides aligned for the consensus sequence (results not shown). To determine whether EVI1 is potentially involved in the direct regulation of miR-138-5p-1 expression in chronic iron-exposed FT194 cells, we reduced EVI1 levels using an siRNA transfection (siB) approach and validated the efficiency of knockdown via western blotting (Fig. 7b). We then determined the levels of miR-138-5p via real-time PCR analysis. Although we confirmed that the levels of miR-138-5p were reduced following chronic FAC exposure (*p* < 0.0001), as noted previously (see Fig. 6d), its levels were not significantly altered upon siB (EVI1 knockdown) treatment in FAC-exposed FT194 cells relative to FAC-exposed parental cells (*p* = 0.8509) (Fig. 7c). These results indicate that miR-138-5p expression is regulated in an EVI1-independent manner and that other factors likely contribute to the downregulation of miR-138-5p under these conditions.

### miR-138-5p partially regulates transcript expression of the stem cell marker TERT but not ALDH1A2 protein

**Figure 8:**
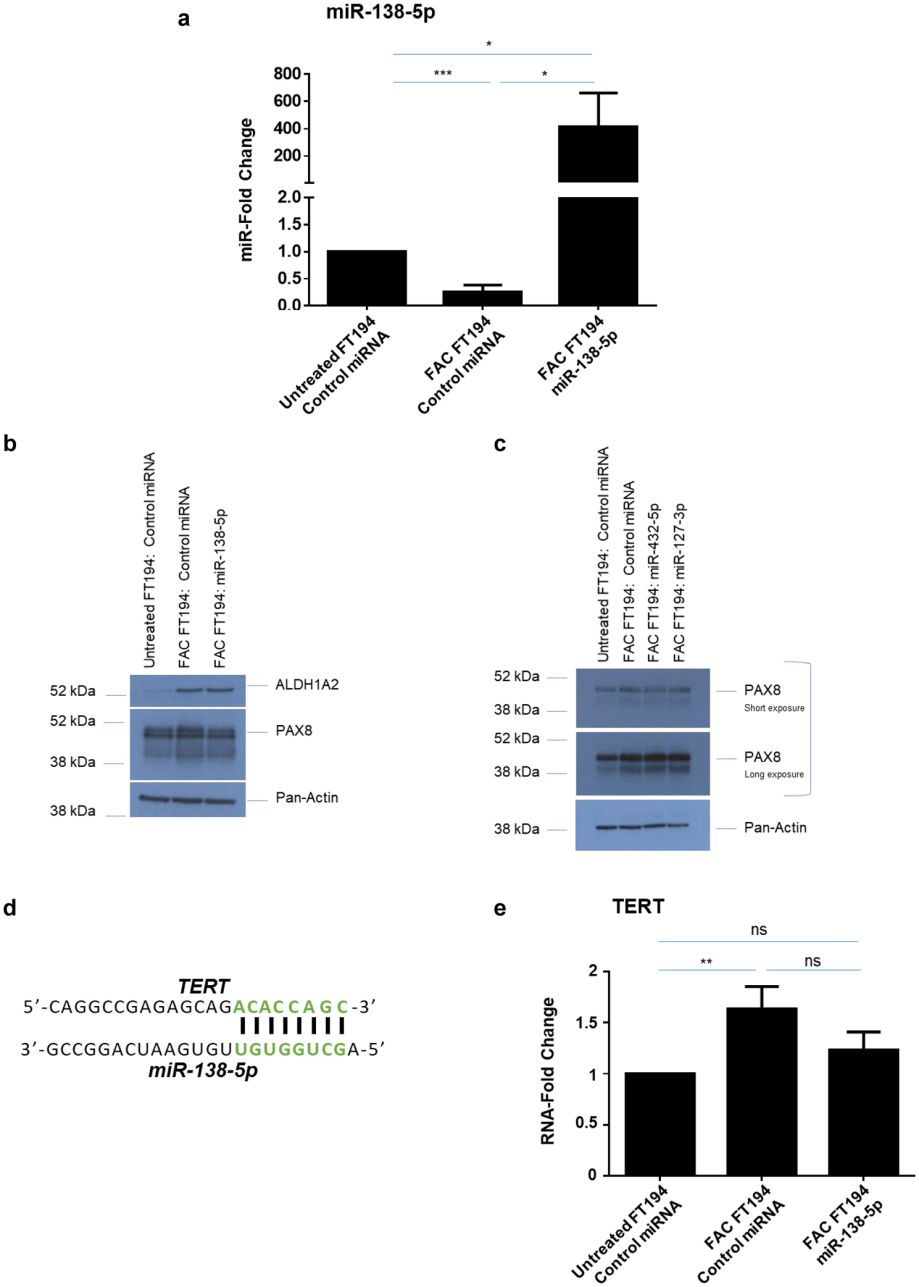
miR-138-5p overexpression partially regulates stem cell marker hTERT transcript levels, but does not alter another stem cell marker ALDH1A2. (**a**) Real-time PCR analysis of miR-138-5p after isolating total miRNAs from miR-138-5p transfected 250 nM FAC-treated cells relative to control transfected FAC-treated and Untreated FT194 cells, at days 122, 125 and 129 of FAC treatment (p = 35, 36, and 37) to validate the overexpression. (**b**) Western blotting was performed for cell lysates collected from miR-138-5p transfected and control transfected Untreated and FAC-treated FT194 cells to analyze ALDH1A2 levels at days 119, 122, and 126 of FAC treatment (*p* = 35–37). White space between cropped blots delineate different antibody applications to the same blot. The full-length uncropped blots are displayed in the Supplementary Information File. (**c**) Western blotting was performed using cell lysates collected from control or miR-432-5p or miR-127-3p, transfected Untreated and FAC-treated FT194 cells with the indicated antibodies at days 123, 131, and 137 of FAC treatment (*p* = 35, 37, and 38). White space between cropped blots delineate different antibody applications to the same blot. The full-length uncropped blots are displayed in the Supplementary Information File. (**d**) The predicted miR-138-5p binding site in the TERT sequence is shown. (**e**) Real-time PCR analysis of TERT in miR-138-5p transfected FAC-treated FT194 cells, relative to control transfected Untreated and FAC-treated FT194 cells after isolating miRNAs at days 119, 122, and 126 of FAC treatment (*p* = 35–37). The data is the composite of three independent experiments.

Cancer stem cells (CSCs or tumor initiating cells) represent a subpopulation of cells that are responsible for tumor initiation and progression^75,76^. Dysregulated iron homeostasis in CSCs may also aggravate cancer phenotypes^77,78^. CSCs are notably characterized by the expression of stem cell markers including elevated activity by aldehyde dehydrogenase (ALDH, represented by multiple isoforms)^79^, a superfamily of metabolic markers which serves as a potential poor prognostic factor of cancer^80^. One family member, ALDH1A2, is aberrantly expressed in acute lymphoblastic leukemia cells^81^ and ovarian cancer^82,83^. Interestingly, the proteomics analyses identified a 3.2-fold increase in ALDH1A2 levels upon FAC exposure in FTSEC cells and IPA analyses suggests that ALDH1A2 is a potential direct target of miR-138-5p (Fig. 5c). Indeed, prior published work supports miR-138-5p involvement in ALDH1A2 regulation^84,85^. To investigate whether ALDH1A2 is a target of miR-138-5p in human FTSECs, we transfected FAC-exposed FT194 cells with miR-138-5p mimic. Although overexpression of miR-138-5p was confirmed by real-time PCR (Fig. 8a) and western blotting demonstrated that ALDH1A2 protein was increased in iron-exposed FTSECs, its levels were not altered by miR-138-5p overexpression (Fig. 8b). These results suggest that miR-138-5p is not a regulator of ALDH1A2 expression in FTSECs. Another stem cell marker TERT^86,87^ has also been reported to be directly regulated by miR-138-5p^74,88^. Since chronic iron exposure can induce TERT mRNA levels^10^ and TERT sequence alignment with miR-138-5p mature sequence has shown direct binding (Fig. 8d)^72^, we analyzed whether TERT increase is mediated via miR-138-5p. Indeed, we observed that miR-138-5p mimic transfection partially rescued the TERT mRNA levels by 25% (Fig. 8e), suggesting that FAC-induced increase in TERT transcript levels could be partially regulated via miR-138-5p.

### miR-432-5p, miR-127-3p, and miR-138-5p overexpression does not alter PAX8 protein but reduces cell numbers in FAC-treated FT194 cells

PAX8 (a member of paired box PAX gene family) is a positive marker of FTSECs^89^ and is elevated in a variety of tumors^90–95^. Our prior findings demonstrated that PAX8 was upregulated following chronic iron exposure^10^; we now validate this observation and further show that it is epigenetically regulated, also supported by prior literature^59^. Furthermore, PAX8 appears to be a common target of miR-432-5p, miR-127-3p, and miR-138-5p via the IPA analyses; target analyses using bioinformatic programs (Targetscan, miRDB, miRNA.org, miRpath v.3 and miRmap) identified PAX8 to be a target of the aforementioned miRNAs in a subset of these databases (data not shown). However, western blot analyses did not identify any marked changes in PAX8 protein expression in FTSECs upon overexpression of these miRNAs (Fig. 8b, c). Thus, it remains to be experimentally determined whether these miRNAs interact directly with PAX8 to regulate its expression.

**Figure 9:**
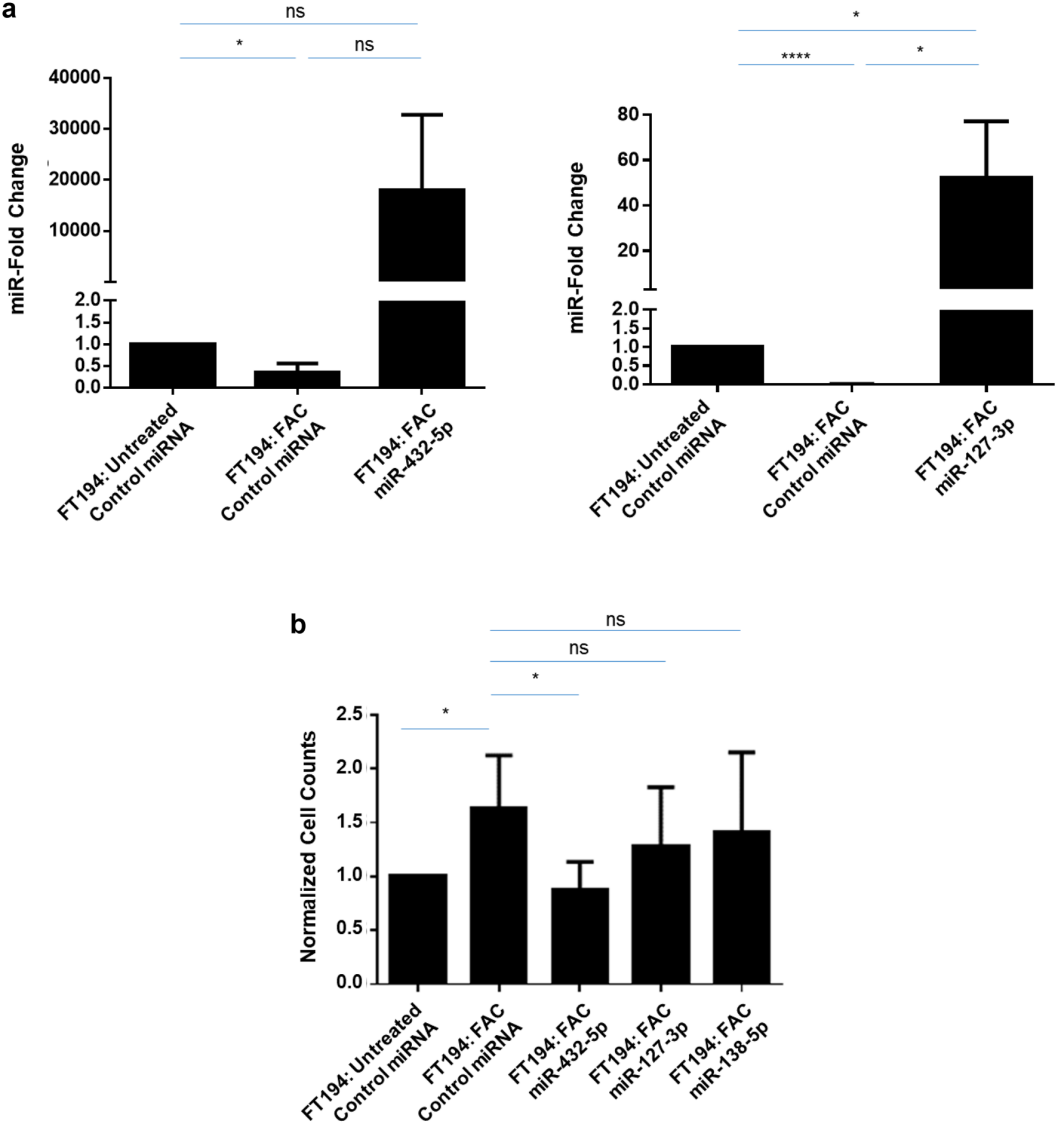
Overexpression of miR-432-5p, miR-127-3p and miR-138-5p in FAC-treated FT194 cells alter the cell counts and Pax8 expression level. (**a**) Real-time PCR of miR-432-5p and miR-127-3p was performed after isolating total miRNAs from the respective mimic-transfected 250 nM FAC-treated cells relative to the Untreated cells at days 123, 131, and 137 with FAC (*p* = 35, 37, and 38). RNU6B was used as a reference control and the data represents a composite of three independent experiments. (**b**) Representation of cell counts obtained from overexpression of miR-432-5p, miR-127-3p, and miR-138-5p compared to control miRNA transfected Untreated and 250 nM FAC-treated FT194 cells. The data presented is the composite of five independent experiments.

Since miR-432-5p, miR-127-3p, and miR-138-5p levels were reduced in chronic FAC-exposed FT194 cells (Fig. 3c–e) and evidence supports their tumor-suppressive role^96–98^, we proposed that rescuing their expression in the chronic iron-exposed FT194 cells could antagonize the FAC-induced increase in cell numbers, as previously reported^10^. Validation of miRNA overexpression for miR-432-5p, miR-127-3p, and miR-138-5p was performed by real-time PCR (Figs. 8a and 9a). Cell counting identified reduced numbers of cells in the chronic iron-exposed FT194 cells, with particular statistical significance following miR-432-5p expression (*p* ≤ 0.05), relative to the parental FAC-exposed FT194 cell line (Fig. 9b).

## Discussion

Iron is an important dietary component that is critical in maintenance of various cellular functions; however, it elicits activity as a mutagenic factor through its participation in Fenton reactions whereby it is involved in generating ROS that may promote DNA damage such as oxidation of DNA bases^99^. In this manner, iron may contribute to the pathophysiology of cancer. Deregulated iron levels and expression of key mediators of iron metabolism are established features of multiple tumors^100^ including an established tumor “addiction” to iron^101^. Indeed, multiple studies have linked the exposure to supraphysiological levels of iron with an increased incidence of cancer including renal tumor formation in a rat model exposed to ferric nitrilotriacetate^102^, iron overload patients (e.g., hemochromatosis) with an increased risk of developing liver tumors^102,103^. On the other hand, dietary iron (in excess) can enhance tumor formation in mice harboring abnormalities in Adenomatous polyposis coli (APC) whereas iron chelation could hinder tumor development^104^. Collectively, these reports implicate the potential tumor promoting activities of iron in various experimental systems.

Although a recent report identified that chronic iron exposure in human pancreatic ductal epithelial cell line supported epithelial-mesenchymal transition (EMT) and tumorigenesis through a p53-dependent mechanism^105^, the role of chronic iron overload in ovarian cancer initiation by mediating transformation of fallopian tube secretory epithelial precursor cells (FTSECs) remains unclear^106^. Iron sources in the pelvic cavity has been suggested to originate from ovulation, retrograde menstrual reflux, and the rupture of follicles^107–109^; furthermore, a link between hemochromatosis and ovarian cancer has been reported^110^. However, there is currently limiting data regarding the contribution of iron to high grade serous ovarian tumor initiation. Recently, we reported that long-term FAC exposure (at 250 nM) to FTSECs leads to cellular changes that are reminiscent of those identified in HGSOC including alterations in EVI1, β-catenin, and c-Myc protein expression, together with functional changes including increased cell numbers and migratory potential^10^. The work reported herein extends these findings to uncover miRNA and protein level changes under these chronic iron exposure conditions in FTSECs.

**Figure 10:**
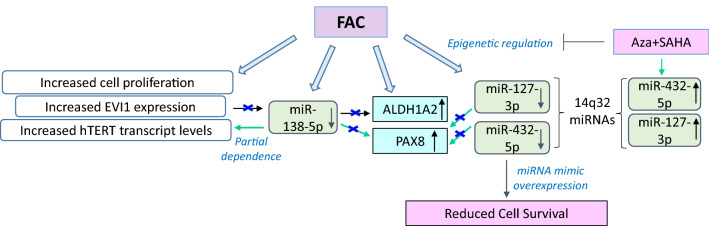
Proposed model for 14q32 miRNA regulation with combination of DNMT inhibition and HDAC inhibition. We previously reported that chronic iron overload contributes towards oncogenic transformative events, which potentially recapitulate early events in HGSOC transformation. Microarray analyses identified markedly reduced levels of miR-432 and miR-127 (located at 14q32) in FAC-treated FT194 cells. We propose that the FAC-induced reduction in the miRNA levels was a result of epigenetic alterations, specifically in their methylation and acetylation status. Inhibition of DNA methyltransferases (using AZA) and HDACs (using SAHA) led to a reversal of the levels of these specific miRNAs in these chronic iron exposed FTSECs. We previously reported that chronic FAC treatment of FT194 cells notably increased TERT mRNA levels^10^ as well as ALDH1A2 protein (from our proteomic analyses, validated via western analysis). We now identified that overexpression of miR-138-5p (located at 3p21) could antagonize the FAC-induced TERT transcript levels; however, miR-138-5p expression did not modulate ALDH1A2 protein. The above described three miRNAs also commonly alter Pax8 protein expression, which may contribute to FAC-induced changes in FT194 cells. Green arrows denote partial regulation.

Further, it is well recognized that aberrant expression of miRNAs is a characteristic of ovarian tumors with potential to serve as biomarkers and/or aid as potential diagnostic tools^41,111,112^; however, the contribution of iron in altering their expression has not yet been reported in precursors to ovarian tumors. Thus, to comprehensively assess the iron-induced molecular changes in FTSECs, we performed an integrated miRNA and protein analysis approach in chronic iron-exposed and transformed FTSECs. To our knowledge, this is the first study to utilize a multi-omics approach to assess miRNA and protein level changes in FAC-exposed and transformed FTSECs. Herein, we have identified a subset of dysregulated miRNAs along with their corresponding protein targets via our integrated experimental approach following chronic iron exposure in FTSECs. Furthermore, we identified that several miRNAs were epigenetically dysregulated which may therefore be potentially associated with the transformative-like alterations observed in FTSECs^10^. Figure 10 displays a proposed model of the findings presented herein.

Analyses of oncogenetically transformed FTSECs (which harbors p53 inactivation, c-Myc^T58A^ mutant, and H-Ras^V12A^ mutant) revealed a higher number of altered miRNAs and protein targets relative to long-term iron exposed cells (see Fig. 2c, d). This suggests that these OCV cells are likely to be more extensively transformed than FAC-treated cells. This is not surprising since iron may only be one of many contributing factors mediating tumorigenesis. Indeed, profound alterations of the cellular regulatory networks may indicate the concept of “multiple events” needed to promote propagation of the tumor^113^. Our work presented herein encourages future investigations, including 3-D organoid culture and in vivo mouse xenografts to further explore the contribution of iron dysregulation to tumor initiation and/or the metastatic process. Two-dimensional in vitro culture models lack the complexity of the tumor microenvironment and thus limit physiological relevance^114^. Although murine models have been developed to investigate HGSOC pathophysiology^115,116^, these in vivo model systems pose challenges in terms of studying iron derived from the reproductive organs due to a lack of menstruation in mice. Therefore, alternative strategies such as intra-bursal or intraperitoneal delivery using iron dextran may be needed. Three-dimensional organoid cultures may also provide an alternative approach to investigate the response of FTSECs to exogenous iron^117^. However, our initial attempts to maintain our oncogenically transformed and chronic iron exposed FTSECs in spheroid culture failed to produce spheroids (data not shown). Thus, implementation of alternative 3-D methods including hanging drop method in future studies could be implemented^118^.

Interestingly, there exists literature supporting the association between iron and miRNAs. In one aspect, miRNAs can behave as iron sensors to suppress expression of proteins associated with iron regulation^119^. On the other hand, the role of iron in deregulating miRNAs is comparatively understudied. Although cytosolic iron can regulate miRNA biogenesis by altering miRNA precursor processing^120^, the role of iron-induced miRNA regulation in cancer requires further investigation. Herein, we have identified global dysregulation of miRNAs following chronic iron exposure. There is evidence implicating iron in the regulation of epigenetic control. Specifically, iron deprivation alters DNA methylation and histone deacetylation in preadipocytes^121^ whereas brain iron overload reduces DNA methylation^57^. However, the specific underlying mechanism through which iron modulates these events remains to be explored. In the microarray analysis presented herein, we identified 20 out of 35 miRNAs (including miR-432-5p and miR-127-3p) that were down-regulated in FAC-treated FT194 cells and also part of a large imprinted miRNA cluster at 14q32 locus. Interestingly, there is evidence implicating epigenetic modification in the regulation of the miRNAs located within this cluster^63,64^. miR-138-5p, although located at a different chromosomal region at chromosome 3p21, was also identified to be markedly down-regulated following iron exposure. Current evidence supports miR-138-5p as a tumor suppressor in various cancer types^88,122–124^. This locus harbors a tumor suppressor gene cluster comprised of 8 genes within a ~ 120 Kb spanning region^125^ whose expression can be regulated epigenetically^126^. Besides miR-138-5p, 4 miRNAs (miR-135a-1, miR-let-7 g, miR-1226, miR-564) out of total 16 miRNAs at this locus have been reported to perform tumor suppressive functions in various cancers^127–131^. Furthermore, upregulation of six miRNAs has been identified in different cancer types, including miR-4271^132^, miR-191^133–135^, miR-425^136–138^, miR-4793^139^, miR-2115^140^, and miR-4443^141^, suggesting potential oncogenic functions in these cancers.

The miR-138-5p down-regulation was partially reversed via inhibition of methylation and histone deacetylases, suggesting that iron can regulate miRNA expression by mediating epigenetic changes in FTSECs. Out of the 15 miRNAs (from a total of 35 miRNAs) identified from the integrated microarray analysis (see Table 1) that are not located at 14q32, 9 are reported to be epigenetically regulated (miR-34a^25,142^, miR-145^143^, miR-182^144^, miR-31^145^, miR-708^146^, miR-34c^147^, miR-125b^148^, miR-615^149^, and miR-17^150^). Similarly, miR-17, which is a part of the 17–92 miRNA cluster located at chromosome 13q31, can also be regulated by epigenetic mechanisms^150^. We also identified 5 miRNAs at chromosome 19q13 to be altered in OCV transformed FT194 cells (relative to CV) via proteomics analysis. Notably, chromosome 19 contains a large imprinted miRNA cluster comprising 46 miRNAs, which can also be regulated via epigenetic mechanisms^151,152^. Collectively, our miRNA analyses have verified epigenetic regulation as a common mechanism underlying miRNA expression, which is also supported by literature for miRNA cluster regulation at different loci in the human genome.

As shown in Fig. 6b, inhibition of methylation and histone deacetylation rescued the levels of miR-432-5p and miR-127-3p in chronic iron exposed FTSECs. This suggests that iron may regulate the activities of epigenetic regulators. There is also evidence that differential miRNA regulation may occur within this locus as independent miRNA regulation of 14q32 at unique miRNA promoters by nuclear receptors may be responsible^153^, although there is also evidence supporting transcription of this miRNA cluster as a polycistronic transcript^43^. Since we observed differential expression of 14q32 miRNAs via our microarray analysis, this may implicate independent regulatory mechanisms for multiple miRNAs within this region.

PAX8 (a member of paired box PAX gene family) is a positive marker of FTSECs^89^ and is elevated in a variety of tumors^90–95^. Our prior findings demonstrated that PAX8 was upregulated following chronic iron exposure^10^; we now validate this observation and further show that it is epigenetically regulated, also supported by prior literature^59^. Furthermore, PAX8 appears to be a common target of miR-432-5p, miR-127-3p, and miR-138-5p via the IPA analyses; target analyses using bioinformatic programs (Targetscan, miRDB, miRNA.org, miRpath v.3 and miRmap) identified PAX8 to be a target of the aforementioned miRNAs in a subset of these databases (data not shown). However, western blot analyses did not identify any marked changes in PAX8 protein expression in FTSECs upon overexpression of these miRNAs. Future studies are needed to experimentally validate whether PAX8 is a target of these miRNAs.

Our earlier findings demonstrated that long-term iron exposure in FTSECs leads to altered expression of EVI1 variants^10^. Although we identified an EVI1 binding site within the promoter region of miR-138-5p through our bioinformatics analysis (see supplementary Fig. S5), EVI1 knockdown did not alter miR-138-5p levels implicating the involvement of other regulatory mechanisms.

The stem cell marker, ALDH1A2^80,154,155^ is reported to be a direct target of miR-138-5p, as identified via a proteomics screen in zebrafish embryos^84^ but as of yet has not been confirmed in humans. As shown in Fig. 8b, overexpression of miR-138-5p in iron exposed FTSECs did not result in an alteration in ALDH1A2 expression; thus, this suggests that ALDH1A2 is not regulated by miR-138-5p in FTSECs. However, another stem cell marker TERT^86,87^, which has already been demonstrated to directly regulate miR-138-5p^74,88^, appears to be at least partially regulated by miR-138-5p (see Fig. 8d).

Although the quantitation accuracy of both the miRNA transcriptomic and proteomic datasets was validated through orthogonal methods such as qPCR and western blot analysis of selected targets, the miRNA target filtering through IPA is based on previously experimentally determined interactions or computational prediction (e.g., TargetScan). Potential regulatory miRNAs will need to be validated in future studies based on the expression pairing of miRNAs and proteins relevant to specific cancer-related pathways identified in our high-confidence transcriptomic and proteomic datasets. Further improvement can also be made in the proteomics-based methodology, which includes fractionation of the proteome and application of DIA approaches to enhance proteome coverage. In addition to deeper proteome coverage to reveal further differentially expressed proteins, global-scale phosphoproteomic analysis can be employed to identify altered signaling pathways, collectively providing detailed global-scale insight into changes of the molecular landscape associated with iron exposure and oncogenic mechanisms in FTSECs.

## Conclusion

Overall, the methodological approach utilized herein served to identify miRNAs and protein targets associated with long-term FAC treatment in FT194 cells. To our knowledge, this is the first study elucidating a comprehensive set of alterations at the miRNA and protein levels in chronic iron exposed FTSECs, which may identify pathways that may contribute to increased tumorigenic potential. In future studies, epigenetic mapping of chronic iron-exposed cells could be performed to further our understanding of the changes induced by long term iron treatment in fallopian tube precursors.

## Supplementary Information

Supplementary Information 1.

Supplementary Information 2.

Supplementary Information 3.

## Data Availability

The mass spectrometry proteomics data have been deposited to the ProteomeXchange Consortium via the PRIDE^28^ partner repository with the dataset identifier PXD018416. The RNA microarray data have been deposited to the Gene Expression Omnibus (GEO)^156^ with the dataset identifier GSE150622.

## Abbreviations

ALDH1A2: Aldehyde dehydrogenase 1 family, member A2
AZA: 5′-Azacytidine
CV: Control Virus Infected
DDA: Data-Dependent Acquisition
DMEM/F12: Dulbecco’s Modified Eagle Medium Nutrient Mixture F-12
DMRs: Differentially Methylated Regions
DNA: Deoxyribonucleic acid
DNMT1: DNA (Cytosine-5-)-Methyltransferase 1
EVI1: Ecotropic Viral Integration Site-1
F: Iron-Treated
FAC: Ferric Ammonium Citrate
FBS: Fetal Bovine Serum
FTSECs: Fallopian Tube Secretory Epithelial Cells
HGSOC: High-grade Serous Ovarian Carcinoma
H-Ras: Harvey Rat Sarcoma Viral Oncogene Homology
hTERT: Human Telomerase Reverse Transcriptase
IPA: Ingenuity Pathway Analyses
LFQ: Label-Free Quantification
LC–MS/MS: Liquid Chromatography with Tandem Mass Spectrometry
MAPK: Mitogen-Activated Protein Kinase 1
miR: MicroRNA
MS: Mass spectrometry
NTBI: Non-Transferrin Bound Iron
OCV: Oncogene Cocktail Virus Infected
PAX8: Paired Box 8
PCR: Polymerase Chain Reaction
PR: Phenol-Red
RPPA: Reverse Phase Protein Array
RNU6B: RNA U6B Small Nuclear
SAHA: Suberoylanilide Hydroxamic Acid
SDS-PAGE: Sodium Dodecyl Sulfate Polyacrylamide Gel Electrophoresis
S-Trap: Suspension-Trap
SV40 LTAg: Simian Vacuolating Virus 40 Large T Antigen
U: Untreated
UPLC: Ultra-Performance Liquid Chromatography
