# Supplementary material for: Global miRNA/proteomic analyses identify miRNAs at 14q32 and 3p21, which contribute to features of chronic iron-exposed fallopian tube epithelial cells
Source: Sci Rep. 2021 Mar 18;11:6270. doi: 10.1038/s41598-021-85342-y (PMC7973504; doi:10.1038/s41598-021-85342-y)

Global miRNA/Proteomic Analyses Identify miRNAs at 14q32 and 3p21, Which Contribute to Features of Chronic Iron-Exposed Fallopian Tube Epithelial Cells

Ravneet Chhabra[1], Stephanie Rockfield[1,3], Jennifer Guergues[1,2], Owen W. Nadeau[2], Robert Hill[1], Stanley M. Stevens Jr. [1,2], Meera Nanjundan[1#]

[1]Department of Cell Biology, Microbiology, and Molecular Biology, University of South Florida, Tampa, FL, USA, 33620

[2]Albany College of Pharmacy and Health Sciences, Department of Pharmaceutical Sciences, 261 Mountain View Drive, Colchester, VT, USA, 05446

[3]Department of Cell and Molecular Biology, St. Jude Children's Research Hospital, 262 Danny Thomas Place, Memphis, TN, USA, 38105

*Correspondence to: mnanjund@usf.edu

Supplementary Figure Legends

Figure S1. Protein targets of miR-138-5p predicted via IPA analysis. IPA analysis identified 28 upregulated proteins predicted to be downstream of miR-138-5p in 250 nM FAC-treated FT194 cells relative to Untreated. Of these, 7 (including ALDH1A2) were related to gynecological cancers. For detailed legend for all molecular processes in the protein network, refer to supplemental Fig. S6.

Figure S2. Protein targets of miR-127-3p and miR-432, associated with malignant solid tumors, predicted via IPA analysis. IPA analysis identified 10 upregulated proteins predicted to be downstream of either miR-127-3p (5 proteins) or miR-432 (5 proteins) in FT194-OCV cells relative to FT194-CV and associated with malignant solid tumors. For detailed legend for all molecular processes in the protein network, refer to supplemental Fig. S6.

Figure S3. Protein targets of miR-432, associated with binding of DNA, predicted via IPA analysis. IPA analysis identified 5 upregulated proteins predicted to be downstream of miR-432 in FT194-OCV cells relative to FT194-CV. Of these, three were associated with binding of DNA. For detailed legend for all molecular processes in the protein network, refer to supplemental Fig. S6.

Figure S4. Protein targets of miR-127-3p predicted via IPA analysis. IPA analysis identified 5 upregulated proteins predicted to be downstream of miR-432 in FT194-OCV

cells relative to FT194-CV. For detailed legend for all molecular processes in the protein network, refer to supplemental Fig. S6.

Figure S5. Prediction of EVI1 binding with miR-138-5p, using Dialign Genomatrix software. The genomic sequence (5000bp upstream) of the promoter region of miR-138-5p-1 (located at 3p21.32) was obtained from the UCSC Genome Browser (www.genome.ucsc.edu, Human Dec. 2013 (GRCh38/hg38 Assembly). The EVI1 N-terminal Zinc-finger binding, C-terminal Zinc-finger binding and consensus sequences were previously published[30-32]. Prediction of EVI1 binding with miR-138-5p-1 promoter region was completed by aligning the sequences using Genomatix software suite (version 3.11, http://www.genomatix.de/cgi-bin/dialign/dialign.pl), and the aligned region is presented.

Figure S6. Detailed figure legend for Fig. 4D, Fig. 5A – 5D, and supplemental Fig. S1 - S4 provided by the Qiagen IPA Software Suite. The prediction legend (on left) and molecular shapes (on right) indicate the colors, patterns and shapes depicting protein measurements and alterations in molecular pathways associated with specific cellular functions. Upregulation and downregulation of targets is denoted by red and green, respectively, with the intensity of color demonstrating the level of change. Arrows indicated the prediction of activation (orange), inhibition (blue), inconsistent findings of downstream molecule (yellow) and no effect (black) while the different molecule shapes represent the type of protein that was expressed or predicted.

Supplementary Tables 1 and 2. Out of the total 4,402 proteins, 3,968 quantifiable proteins were identified after filtering. In OCV-infected compared to CV-infected FT194 cells, out of 4,691 total proteins, 4,148 quantifiable proteins were identified after filtering. Using Welch's t-test ($p < 0.05$) and z-score (z-score >1), statistically significant targets were identified that had LFQ intensity ratio of ≥ 2 or ≤ 0.5. Thus, 622 targets for OCV (relative to CV) and 243 targets for FAC-exposed FTSECs (relative to Untreated) were obtained. These tables show a raw list of total quantifiable as well as differentially expressed proteins.

Additional Information for Western Blots

Uncropped images are displayed for all western blots presented in the manuscript including Fig. 4C, Fig. 6A, Fig 7B, Fig. 8B, and Fig. 8C. Cropped blots are presented in the main figure file; white space in-between each blot represents different antibody applications to the same blot.

Supplemental Figure S1

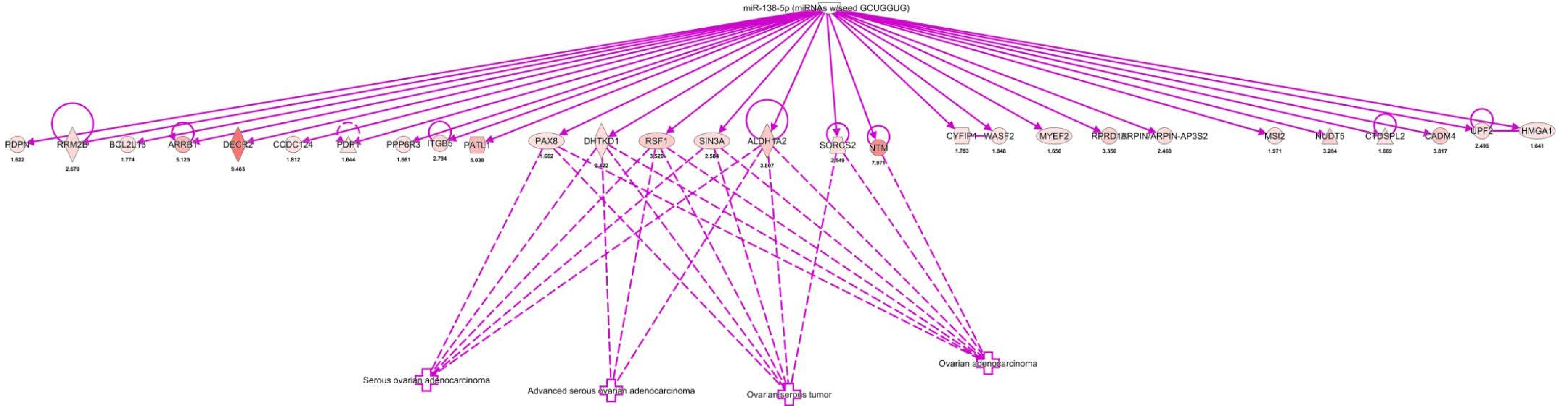

Supplemental Figure S2

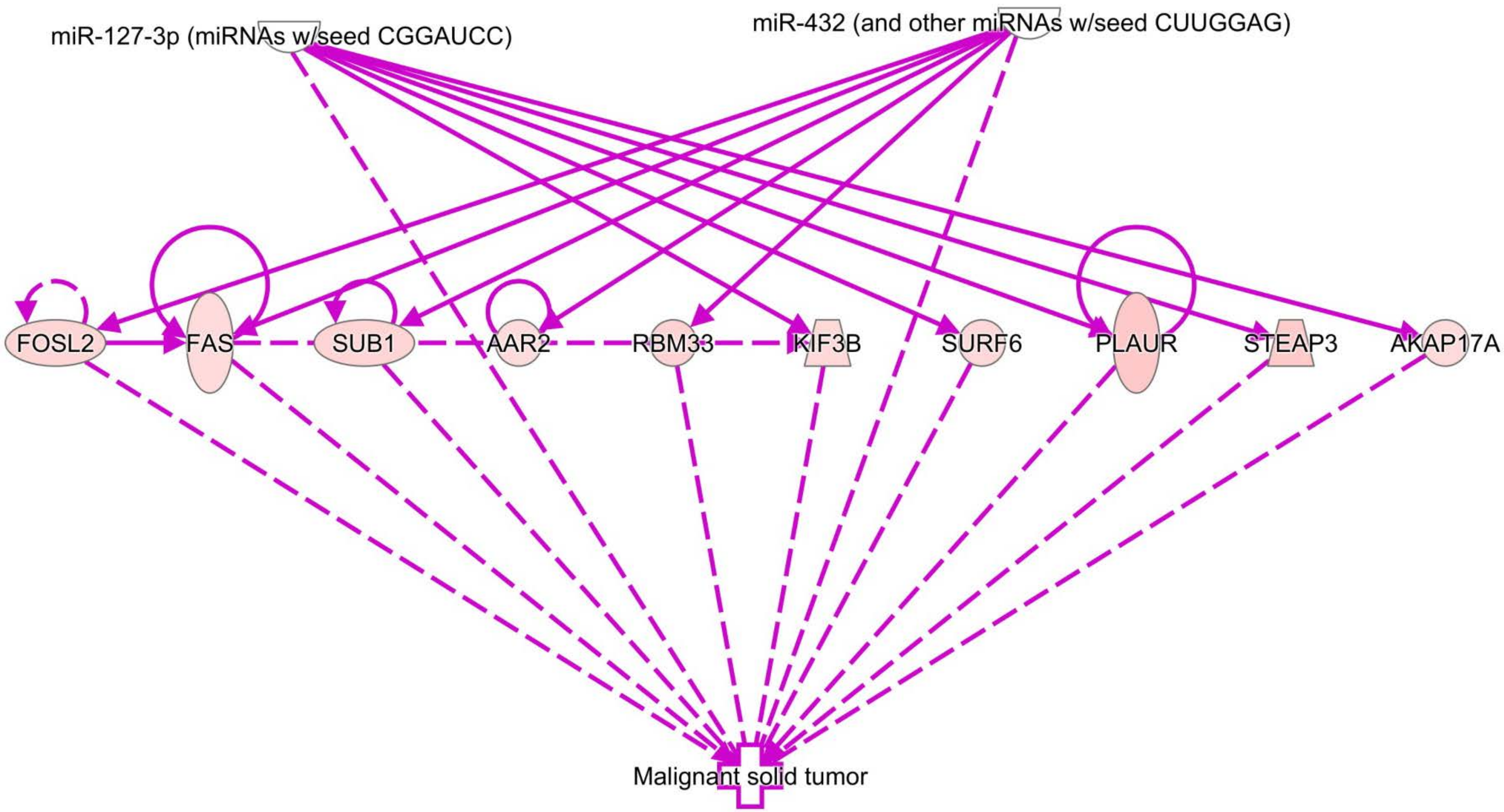

Supplemental Figure S3

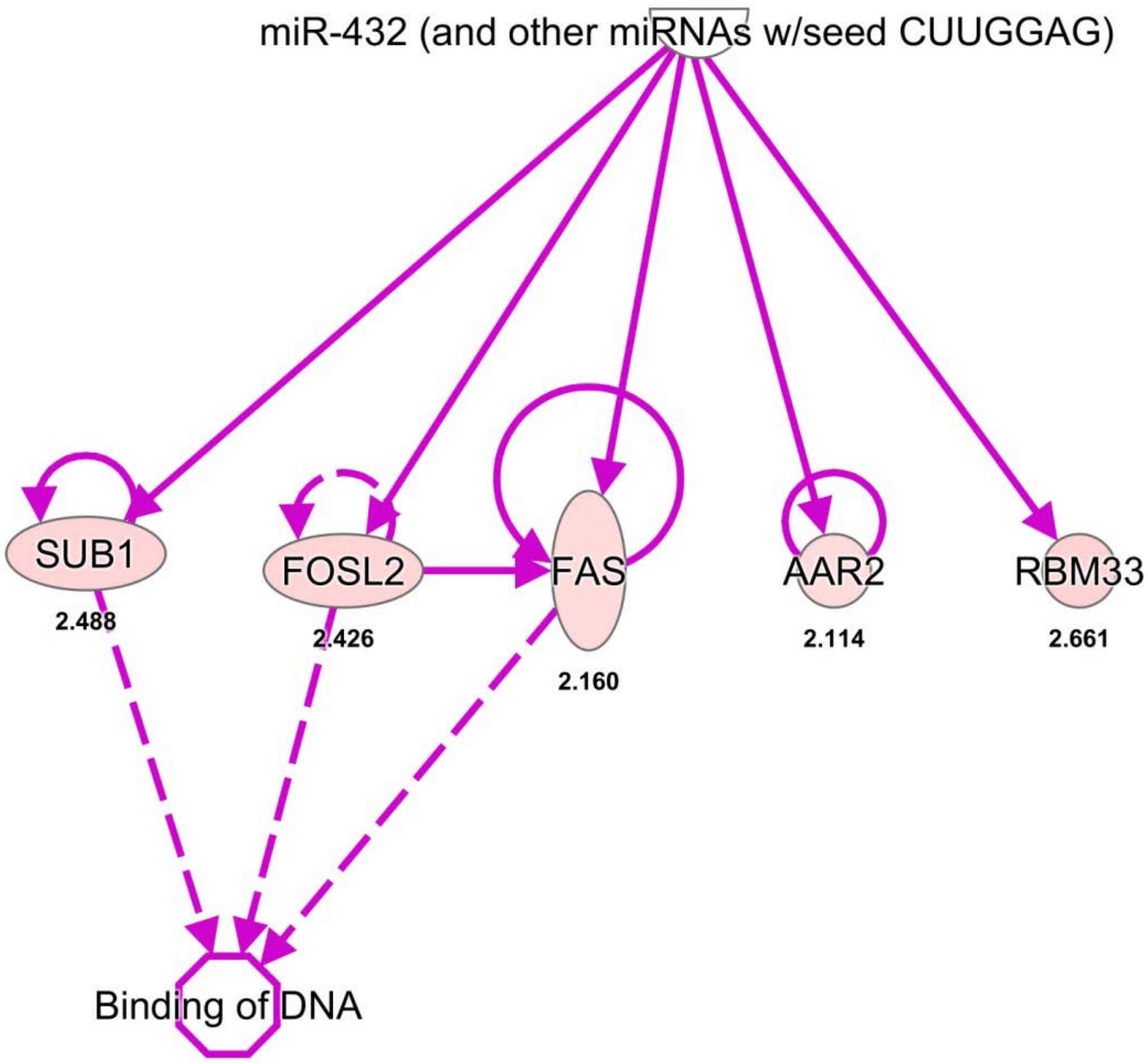

Supplemental Figure S4

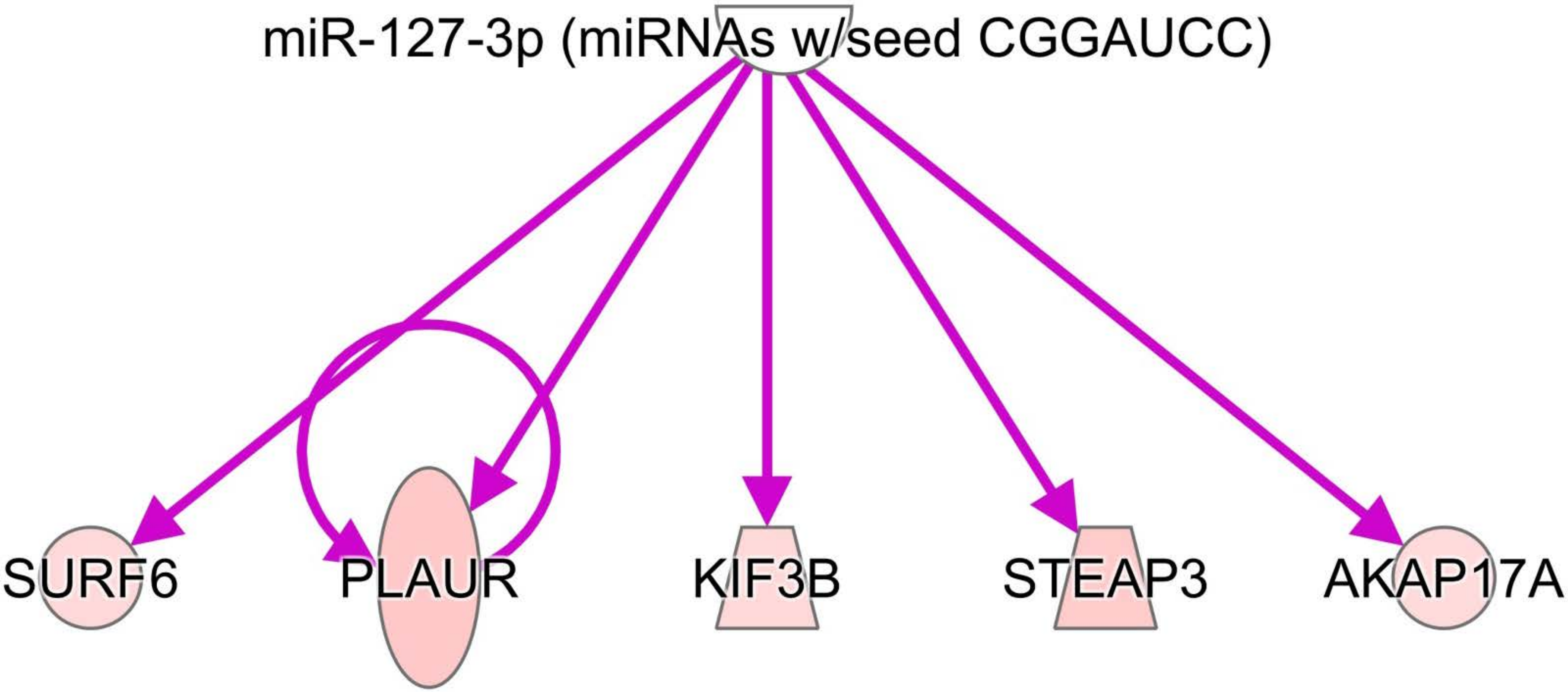

Supplemental Figure S5

```
alignment position                    2521...... 2531...... 2541...... 2551...... 2561...... 2571...... 2581......

Promoter_miR-138           2521   tagcctagag tcagGAAGAT GAGttaagag gtctgacagt ctattgaagt aaaattattc attacagaat
EVI1_N-term_Zn_Finger_BD  4   ---------- -----AAGAT A--------- ---------- ---------- ---------- ----------
EVI1_C-term_Zn_Finger_BD  1   ---------- ----GAAGAT GAG------- ---------- ---------- ---------- ----------
EVI1_Consensus            5   ---------- -----AAGAT Aa-------- ---------- ---------- ---------- ----------
```

Supplemental Figure S6

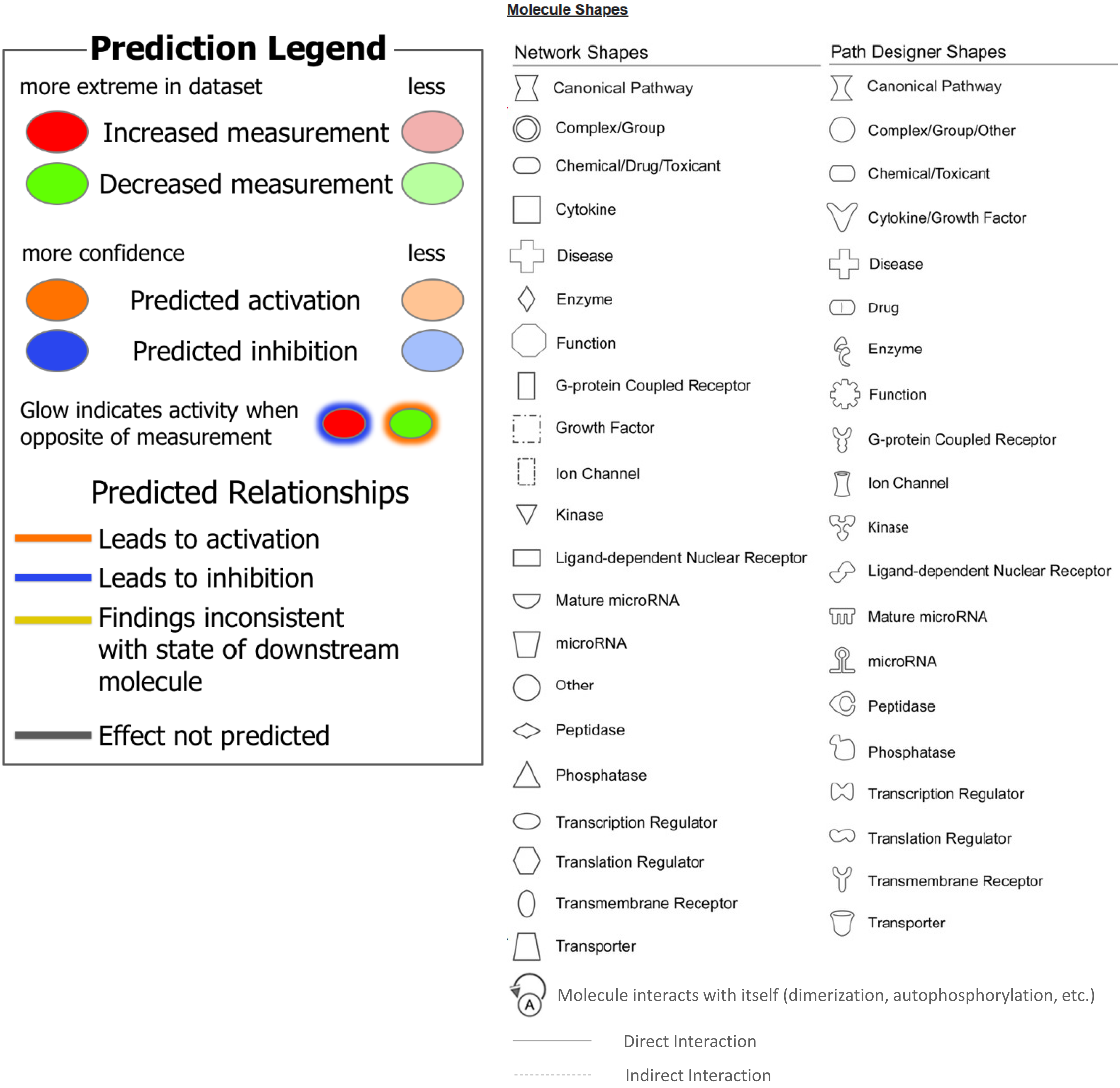

Additional Information for Western Blots

Uncropped -
Fig. 4C, Fig. 6A, Fig, 7B, Fig. 8B, and Fig. 8C

Figure 4C: Replicate #2 (Left Side)

CRYAB

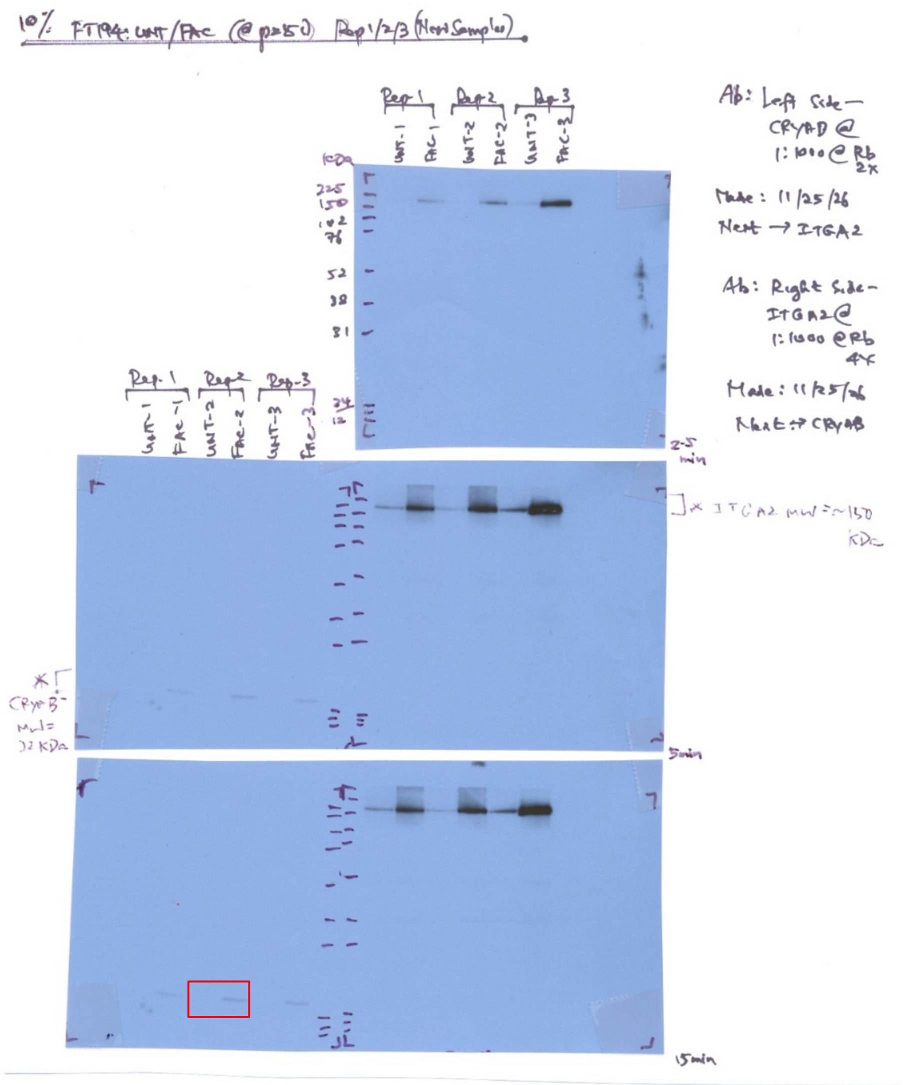

ITGA2

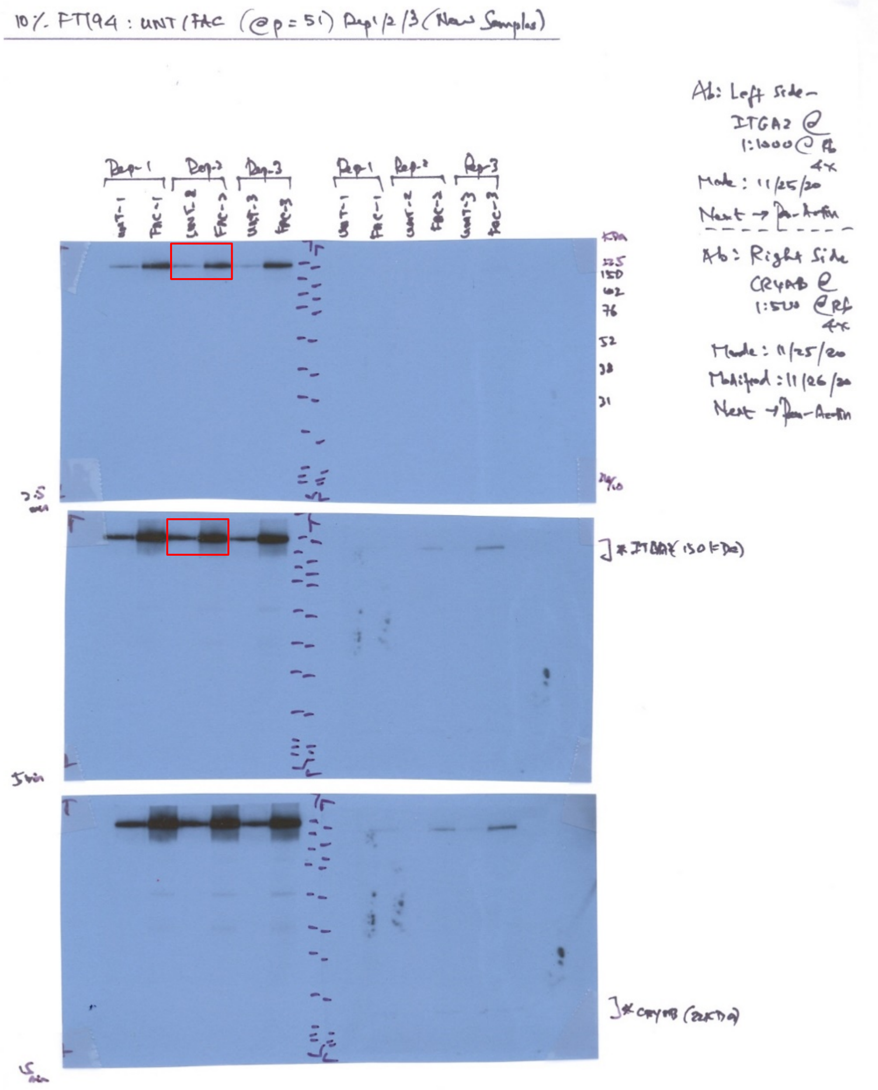

Figure 4C: Replicate #2 (Left Side)

Pan-Actin

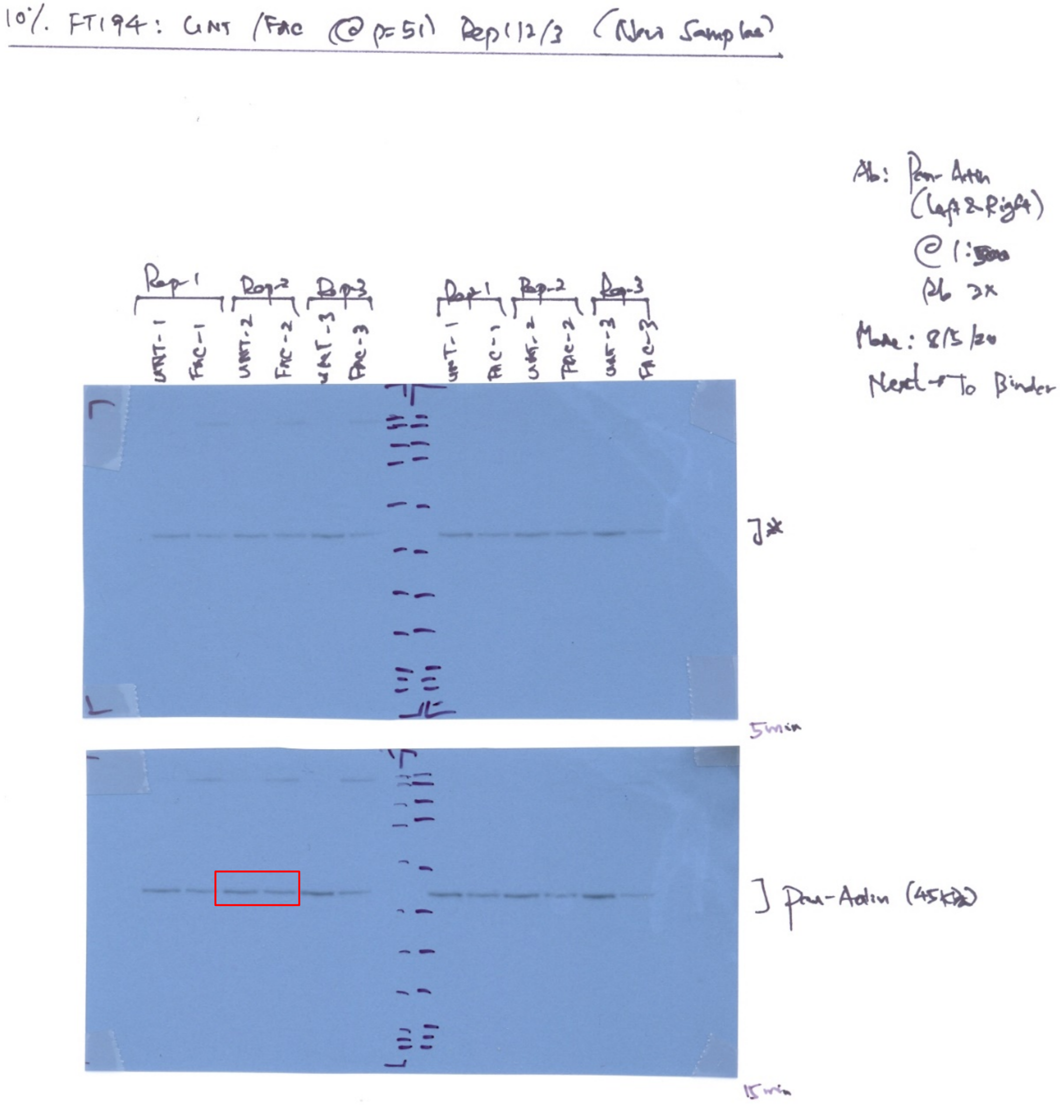

Figure 6A: Replicate #3

Acetyl-H3

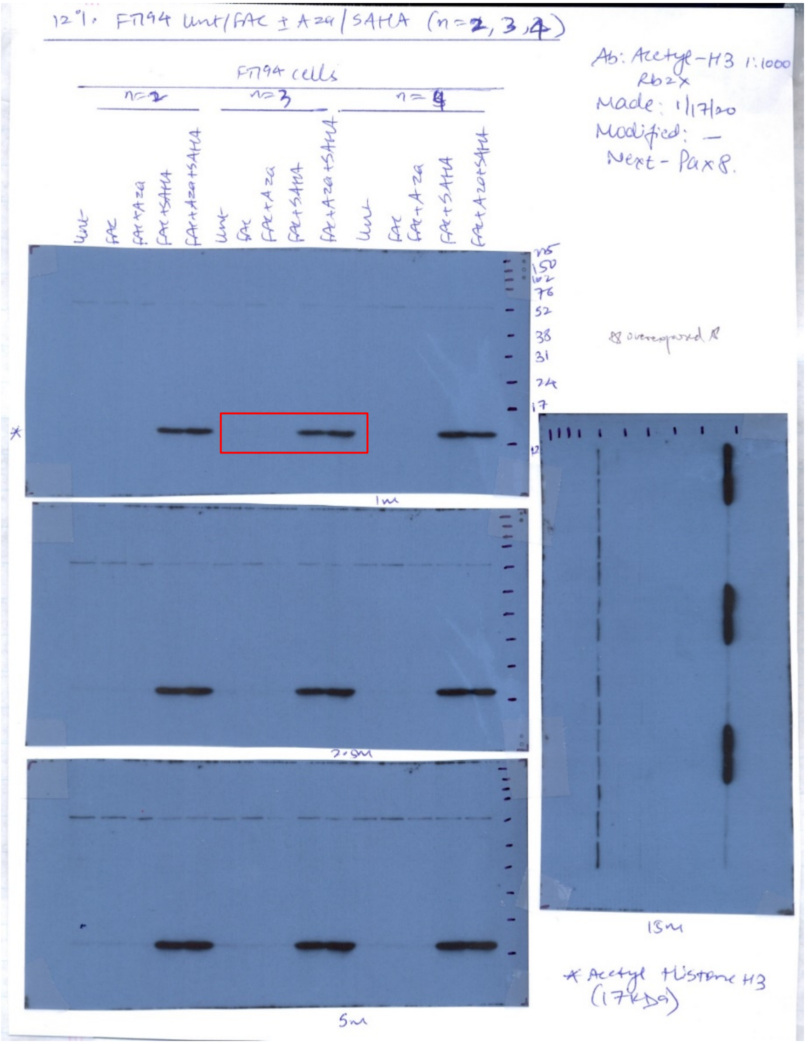

DNMT1

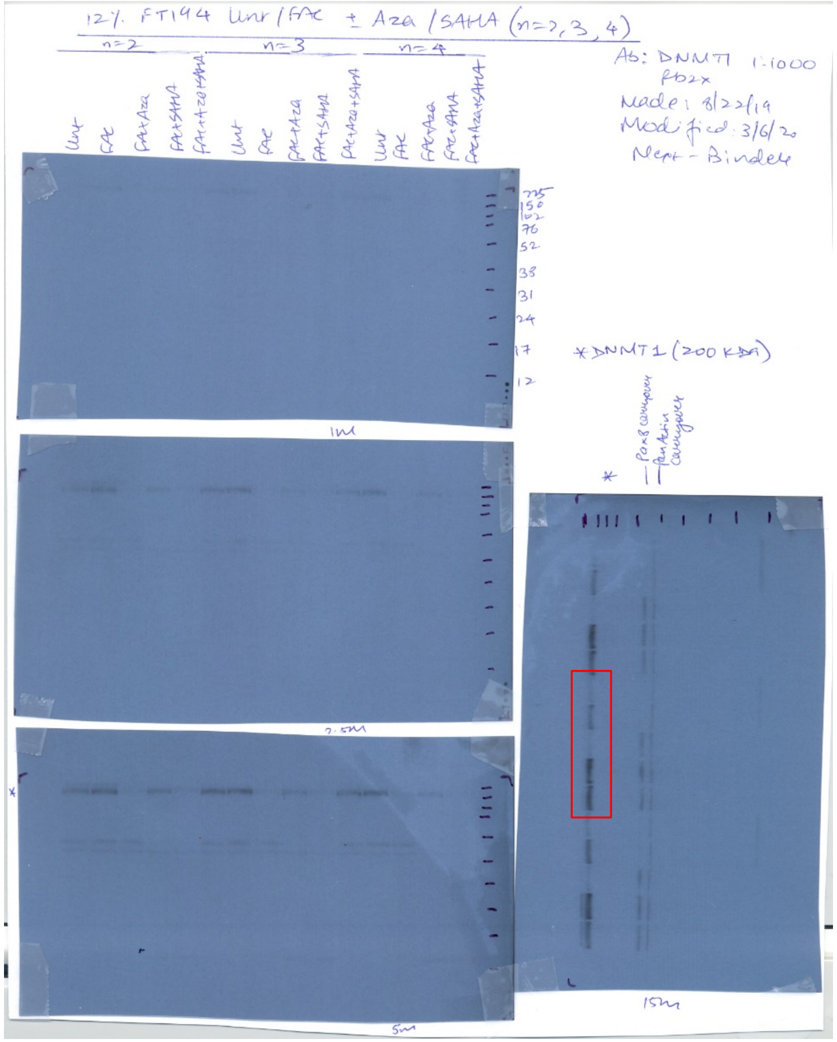

Figure 6A: Replicate #3

PAX8

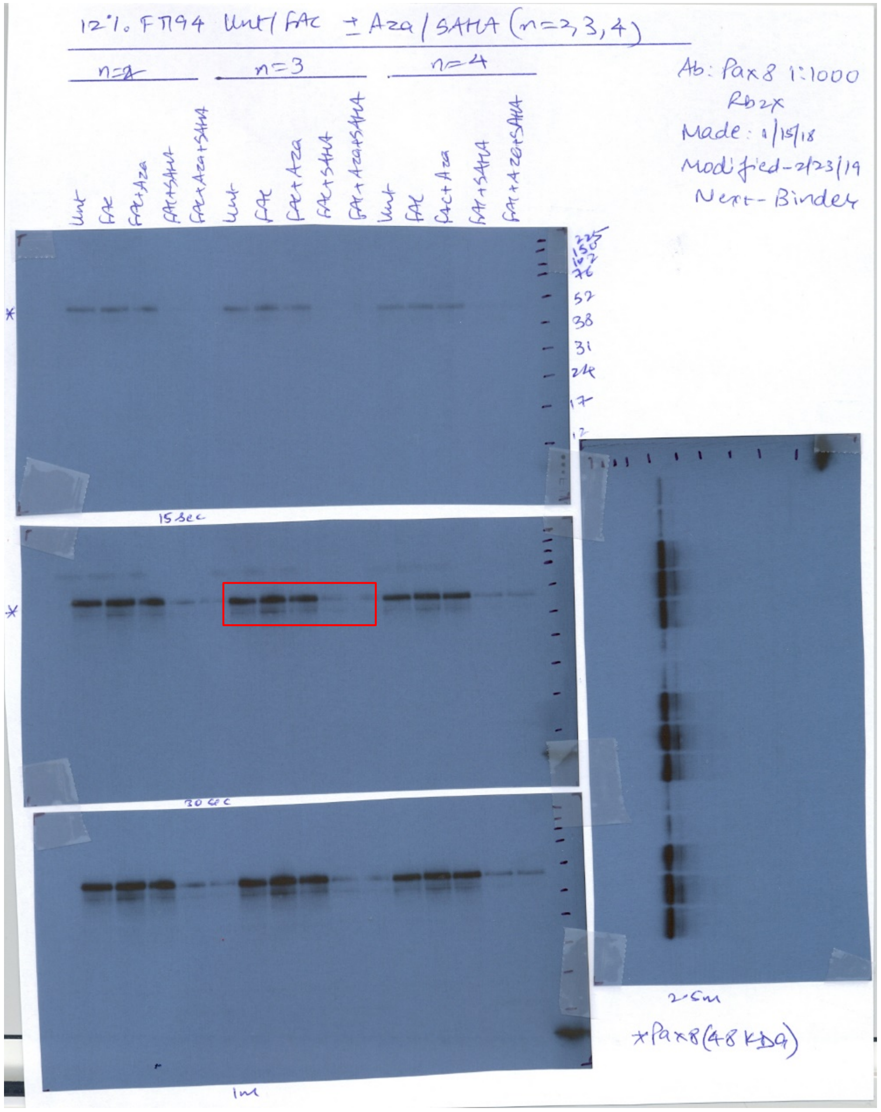

Pan-Actin

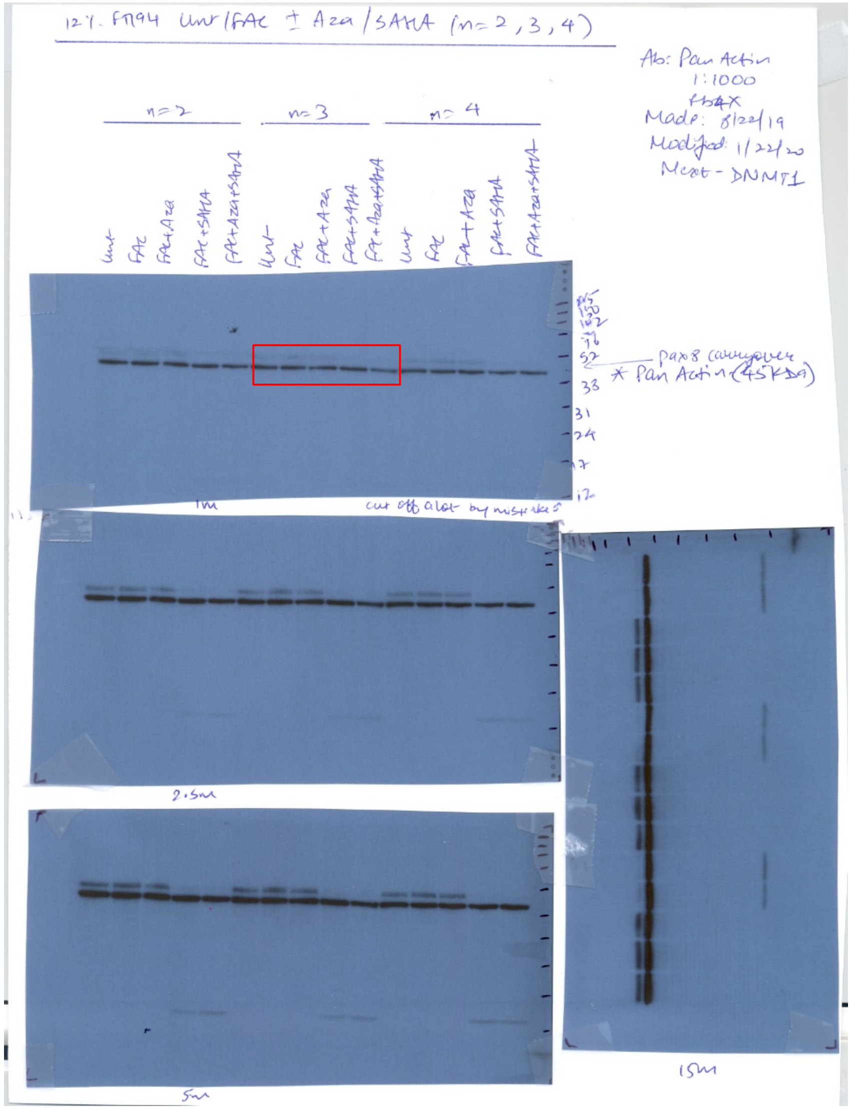

Figure 7B: Replicate #3 (Left Side)

EVI1

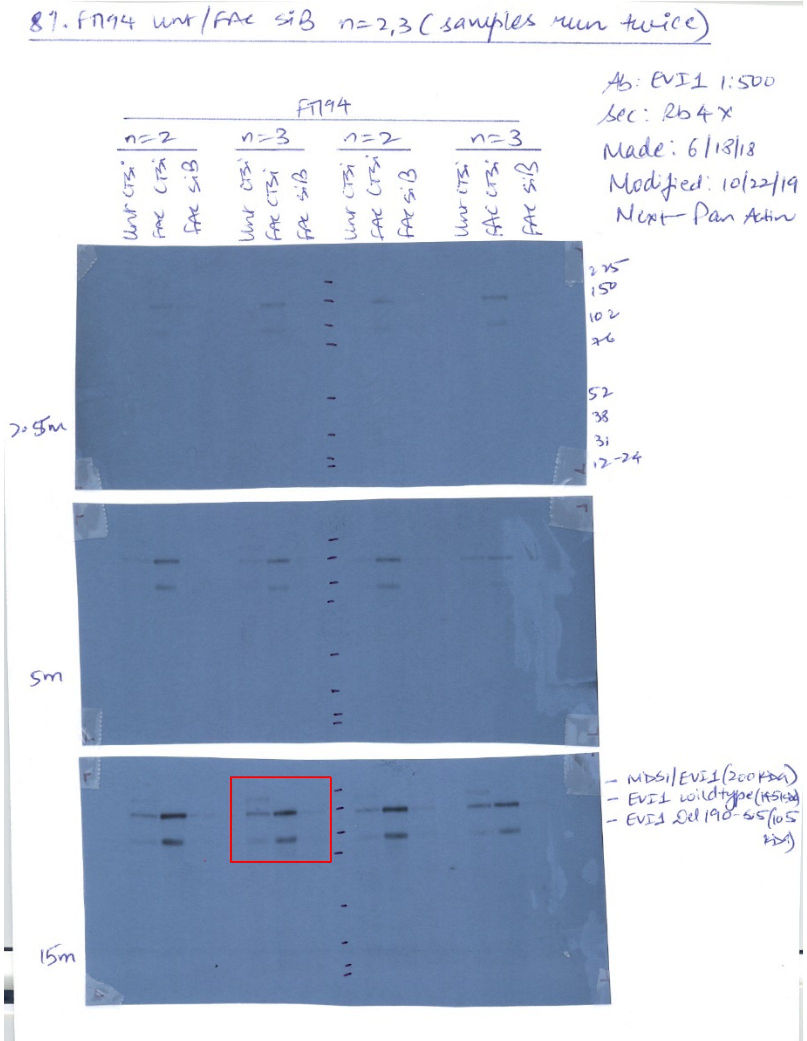

Pan-Actin

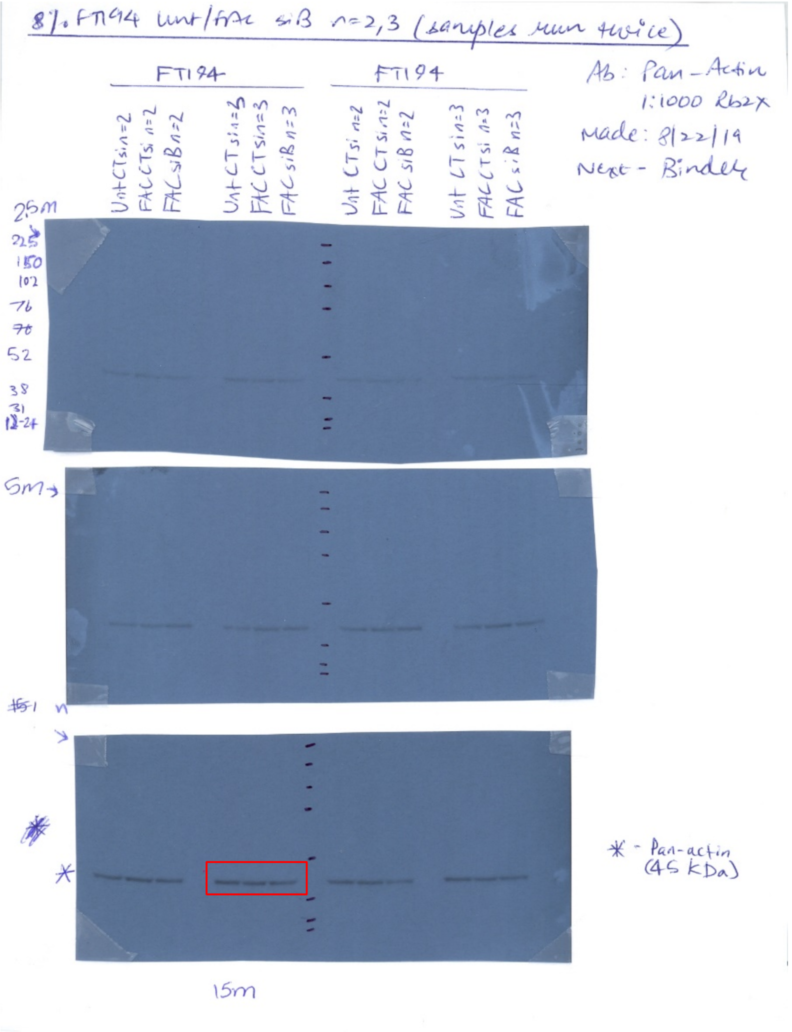

Figure 8B: Replicate #2

ALDH1A2

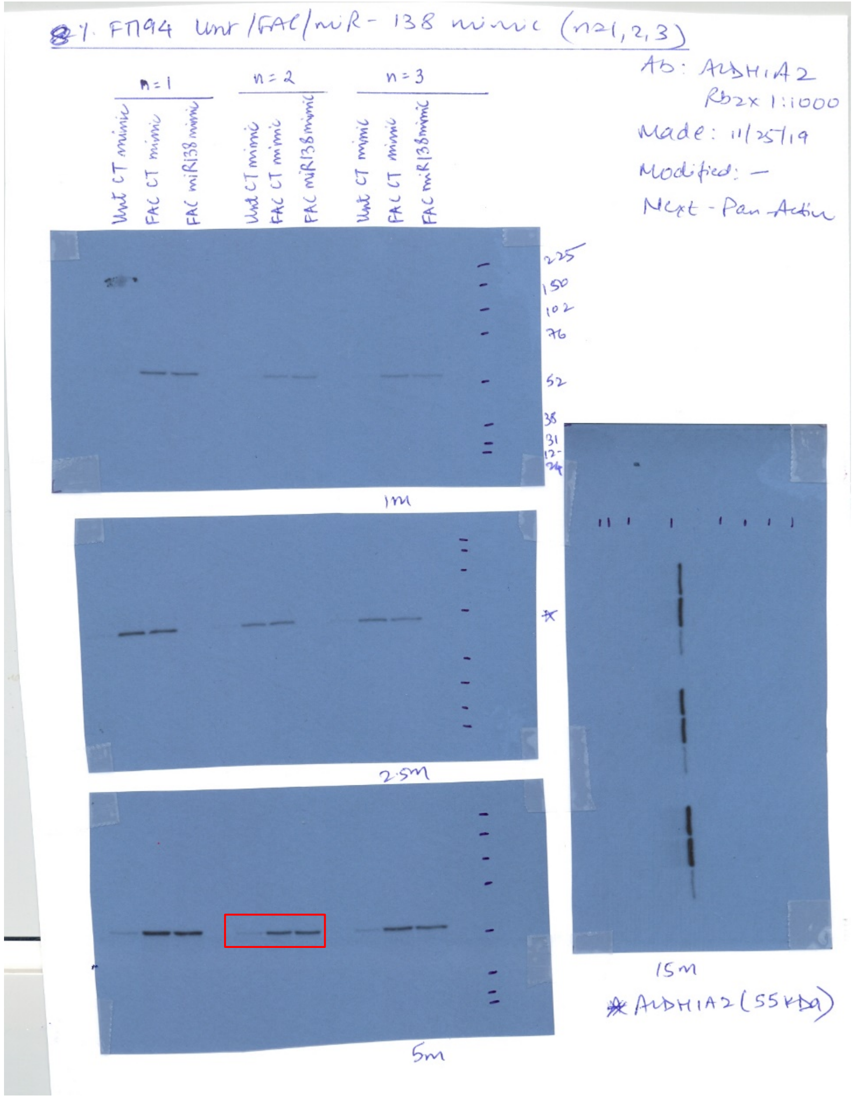

PAX8

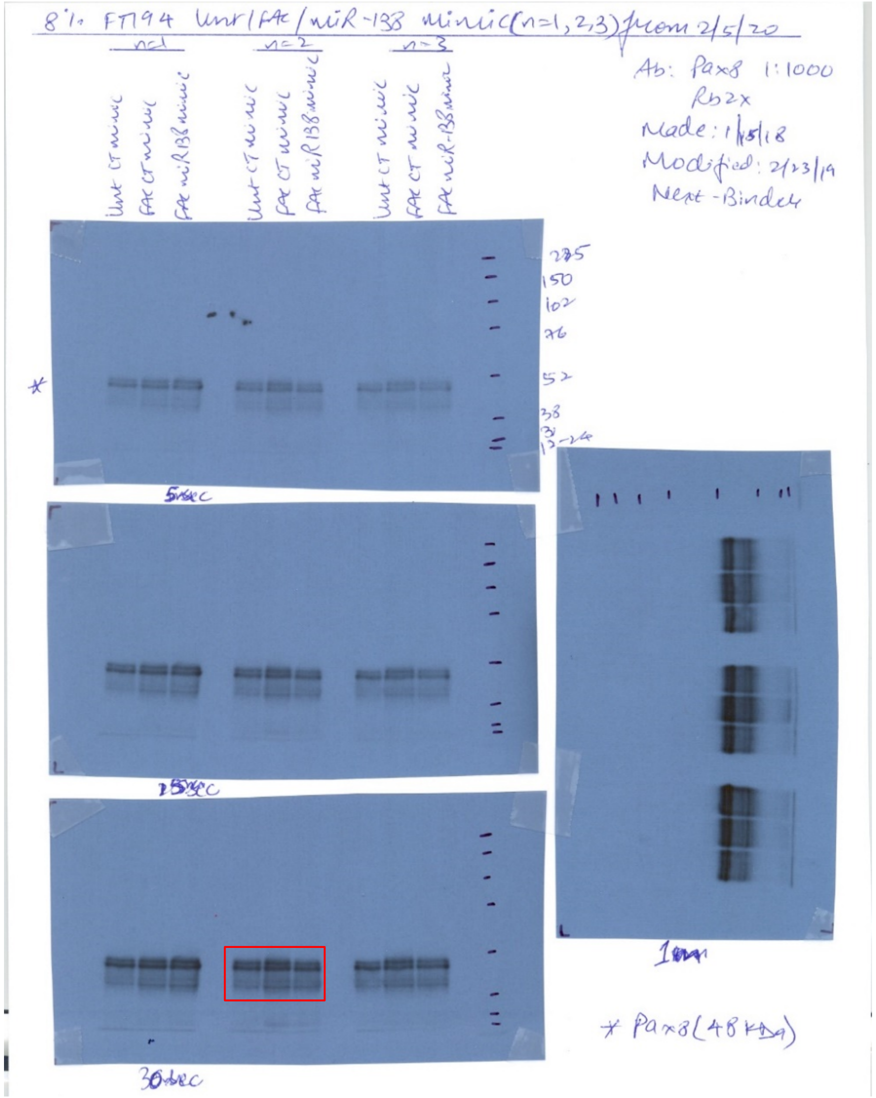

Figure 8B: Replicate #2

Pan-Actin

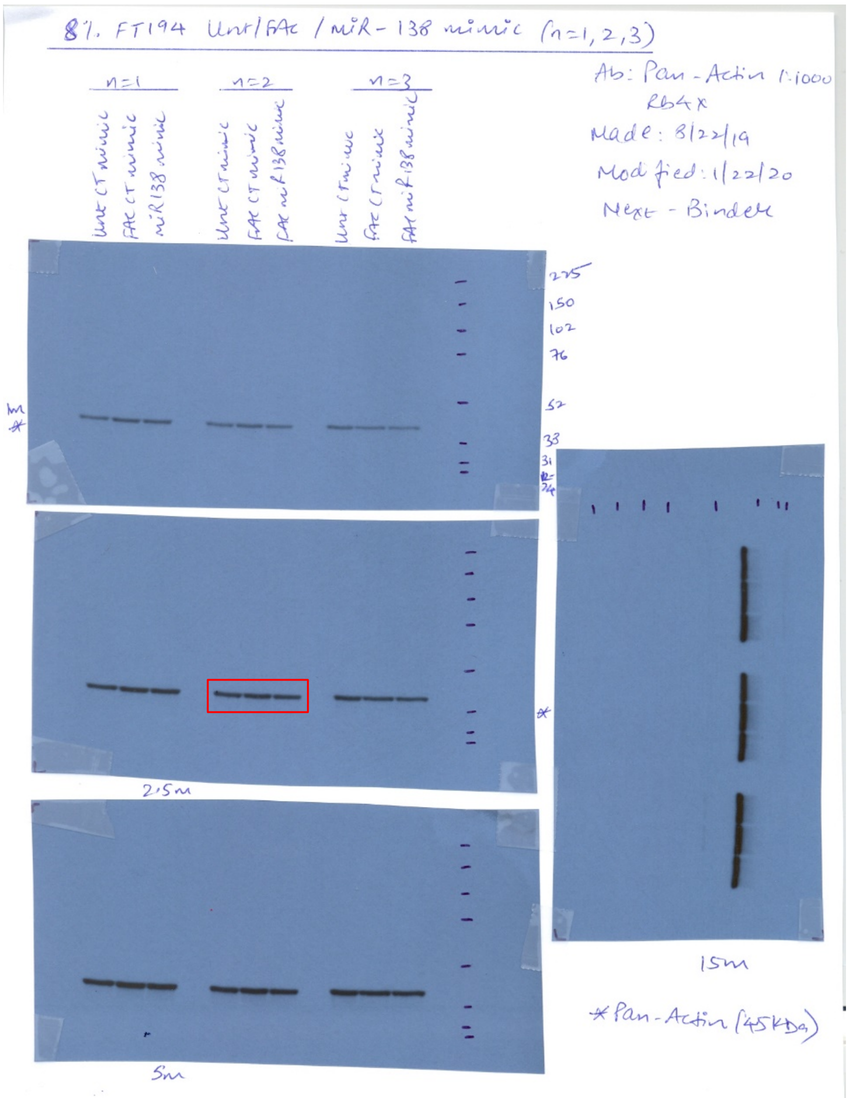

Figure 8C: Replicate #2

PAX8

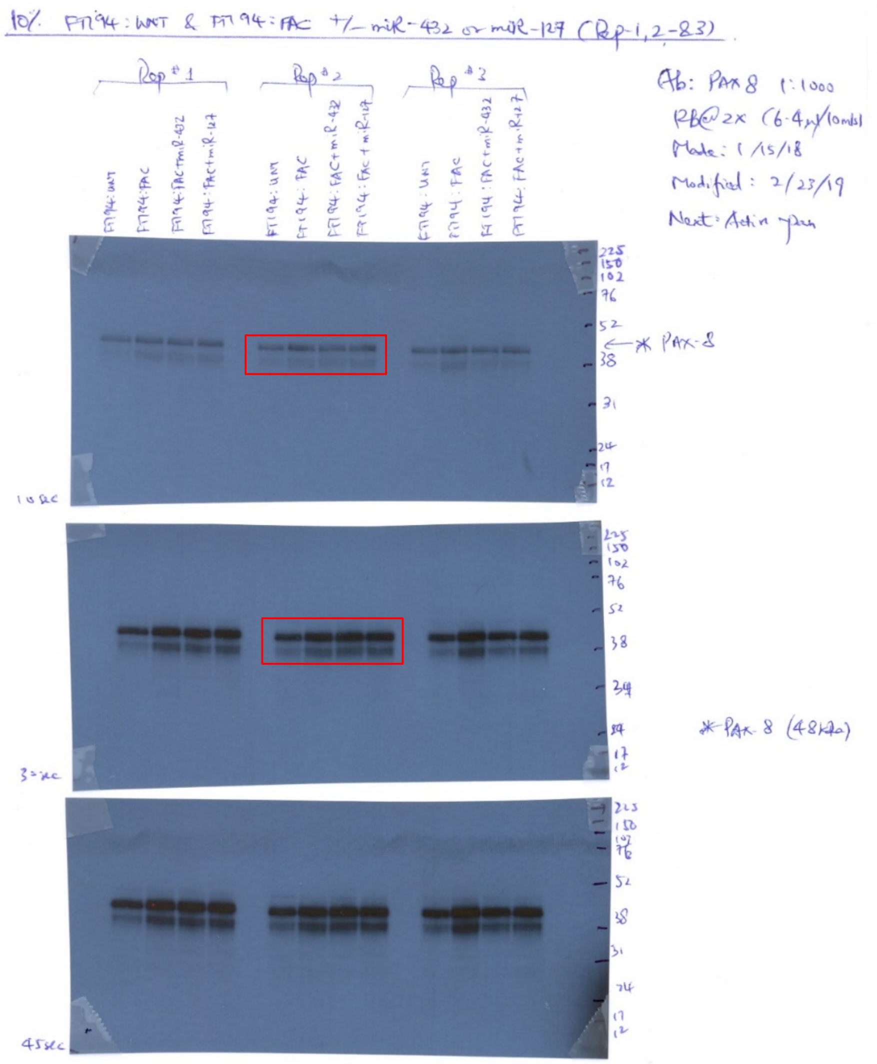

Pan-Actin

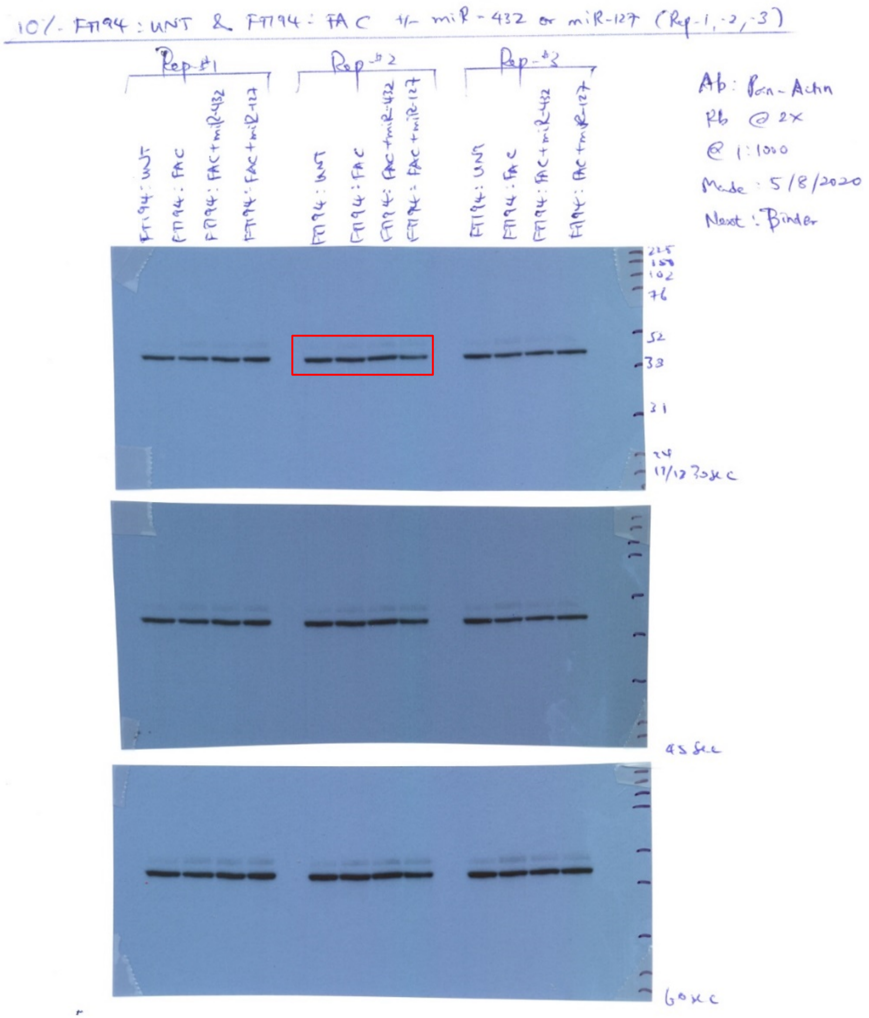

Supplement: Supplementary file 1 — Supplementary Information 1. [file 41598_2021_85342_MOESM1_ESM.pdf]
